# Advancing immune profiling in colon cancer through enhanced lipidomics of low‐input samples

**DOI:** 10.1002/ctm2.70399

**Published:** 2025-07-11

**Authors:** Karim Pérez‐Romero, Cristina Huergo‐Baños, Albert Maimó‐Barceló, Lucía Martín‐Saíz, Teresa Ximelis, Catalina Crespí, Marco A. Martínez, Paloma de la Torre, Myriam Fernández‐Isart, Daniel H. Lopez, José Andrés Fernández, Ramón M. Rodríguez, Gwendolyn Barceló‐Coblijn

**Affiliations:** ^1^ Institut d'Investigació Sanitària Illes Balears (IdISBa) – Health Research Institute of the Balearic Islands Palma Spain; ^2^ Research Unit of the Hospital Universitari Son Espases Palma Spain; ^3^ Department of Physical Chemistry Faculty of Science and Technology University of the Basque Country (UPV/EHU) Leioa Spain; ^4^ Department of Pathological Anatomy Hospital Universitari Son Espases Palma Spain; ^5^ Department of Gastroenterology Hospital Universitari Son Espases Palma Spain; ^6^ Department of Gastroenterology and General Surgery Hospital Universitari Son Espases Palma Spain

1

Dear Editor,

Considering that up to 50% of stage II–III colon cancer (CC) patients may develop metastasis over time,^1^, ^2^ defining new therapeutic strategies for these patients is critical. Despite the success of immunotherapy to treat various malignancies, the benefits for CC are still limited to a small subset of patients, highlighting the urgent need to identify biomarkers capable of predicting immunotherapy response and resistance. Therefore, a comprehensive characterisation of the tumour microenvironment (TME) and the immune landscape within is essential. While disciplines like transcriptomics or proteomics are actively exploring these aspects, lipidomics remains an unexplored strategy.

Herein, we aimed to outline immune cell identity and metabolic state by focusing on the phospholipid profile. However, the lipid profiling of immune infiltrates remains technically challenging due to limited sample input, a common scenario when investigating in clinical environments, which hinders fundamental insights into immune cell metabolism in a tumour context.^3^ To address this non‐trivial challenge, we developed a novel analytical strategy that combines cell micro‐deposition with mass spectrometry (MS) to achieve comprehensive lipid profiling of as few as 1.5 × 10^4^ cells, a number 1–2 orders of magnitude lower than those commonly used in conventional lipidomic approaches.^4^ This method was applied to both circulating and tumour‐infiltrating immune cells obtained from patients with CC. The compositional information gathered was crucial for the subsequent identification of immune infiltrates directly on CC sections based only on their differential lipidome. Importantly, the latter was established using spatially resolved lipidomic techniques, particularly MS imaging (MSI), thereby preserving TME integrity (Figure 1A).

**FIGURE 1:**
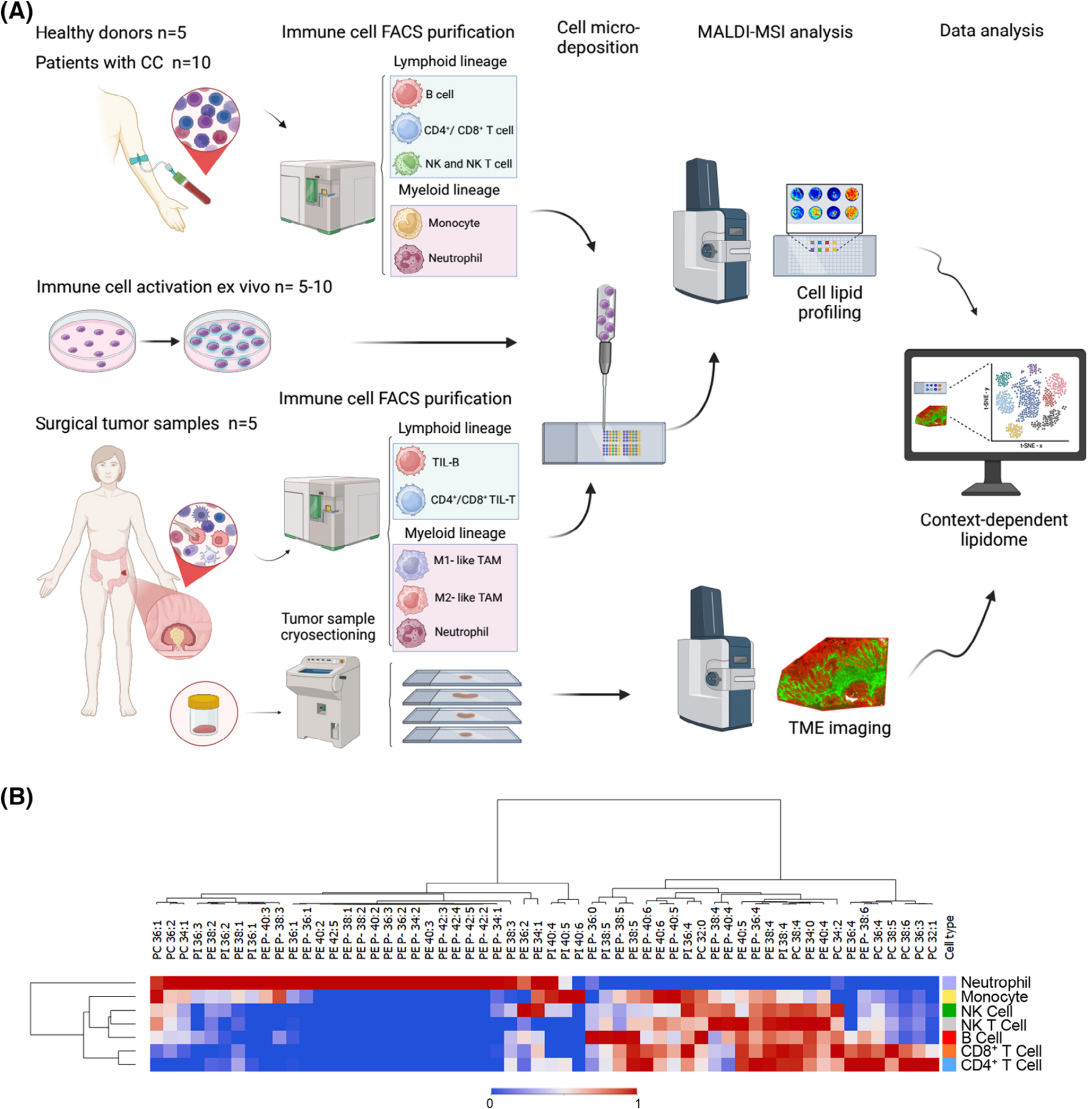
Analytical strategy and lipidomic profiling of immune cells. (A) Overview of the analytical strategy developed for lipidomic profiling using mass spectrometry (MS) techniques, in particular, we used matrix‐assisted laser desorption/ionisation mass spectrometry, which for tissue sections analysis was coupled to imaging. The technique incorporates cell micro‐deposition, enabling the analysis of low‐input immune cell populations, including circulating cells from healthy and colon cancer (CC) donors, as well as tumour‐infiltrating immune cells. (B) Heatmap showing the phospholipid content of isolated circulating immune cells. Relative abundance of individual lipid species is displayed across various immune cell types. Each column represents a distinct lipid species, while each row indicates average abundance per cell type. Data are column‐normalised from 1 (red, higher abundance) to ‐1 (blue, lower abundance), emphasising variations in lipid species abundance between immune cells.

Across the literature, comprehensive information on the lipid composition of immune cell populations, other than lymphocytes and macrophages, is scarce. For this reason, we first isolated and analysed up to seven different immune cell types from healthy donors, which presented a remarkable specificity of the lipidomic profiles (Figure 1B, Figures S1–S3 and Table S1). Cells within the lymphoid lineage (B, T and NK cells) exhibited closer profiles to each other compared to myeloid cells (neutrophils and monocytes), which displayed a wider phosphatidylethanolamine (PE)‐plasmalogens diversity and lower polyunsaturated fatty acid (PUFA)‐species content. Further, monocytes and neutrophils showed remarkably lower PI38:4 content. Hence, these results reinforced the cell‐type‐ and lineage‐specificity of immune cell phospholipid signatures.

Next, we sought to demonstrate whether lipid profiles reflect functional specialisation in response to stimulation by using a comprehensive battery of ex vivo activation conditions (Figure 2A, Figures S4 and S5 and Table S2). Activated T cells exhibited a consistent decrease in arachidonic acid (AA)‐containing species, such as PC36:4, PI38:4 and PE38:4, while B cells maintained a stable profile (Figure 2B). Monocytes (Mo) and neutrophils also displayed changes, but most were not statistically significant (Figure 2B and Figure S6). Conversely, macrophage differentiation led to higher monounsaturated (MUFA) species levels in M1‐like Mo, and higher PUFA species in M2‐like Mo (Figure S7). Overall, ex vivo cell activation induced phospholipid remodelling, affecting the MUFA/PUFA ratio (Figure 2C). These results align with the enrichment in fatty acid metabolism pathways for activated CD4^+^ and CD8^+^ T cells and M1‐like macrophages (Figure S8), based on previously published gene expression data.^5^, ^6^, ^7^

**FIGURE 2:**
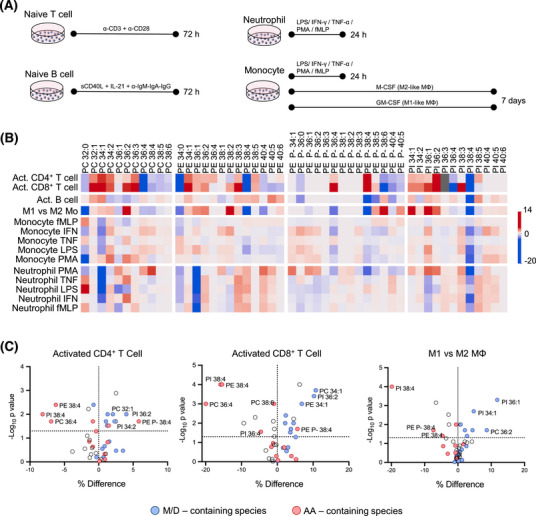
Lipidomic profiling of ex vivo activated immune cell subpopulations. (A) Scheme summarising the main features of immune cell type‐dependent activation protocols. (B) Heatmap showing alterations in phospholipid composition upon ex vivo activation. For simplicity, only lipid species accounting for > 1% of the total lipid class and present in all cell types were included. (C) Volcano plots showing significantly different species in activated CD4^+^ and CD8^+^ T cells, and between M1 and M2 macrophages. Red and blue dots differentiate between arachidonic acid (AA)‐ and MUFA‐species, respectively.

We then investigated the impact of CC on circulating immune cell lipid profiles by analysing neutrophils, monocytes, NK, B, NKT, CD4^+^ and CD8^+^ T cells lipid profiles in patients and healthy donors (Figure 3A, Figures S9–S16 and Table S1). Patients' immune cells showed significant increases in MUFA‐species (34:1 and 36:1) and a consistent decrease of AA‐species, such as PC, PE and PI38:4. Hence, these results demonstrate that peripheral blood immune cells exhibited an activation state phenotype, which is consistent with the chronic tumor‐associated inflammation (elevated pre‐surgery CRP levels > 0.5 mg/dL, Table S3).^8^ Interestingly, we also uncovered a decrease in linoleic acid (LA)‐species (PC34:2, PC36:3 and PE36:2) in all circulating immune cells (10%–30%, depending on cell type, Figure S17), which occurred concomitant to the plasma LA depletion previously reported (>50% decrease).^9^ Hence, these results reinforce the systemic impact of CC and lay the ground to explore phospholipidome sensitivity for early‐stage CC detection or therapy response assessment.

**FIGURE 3:**
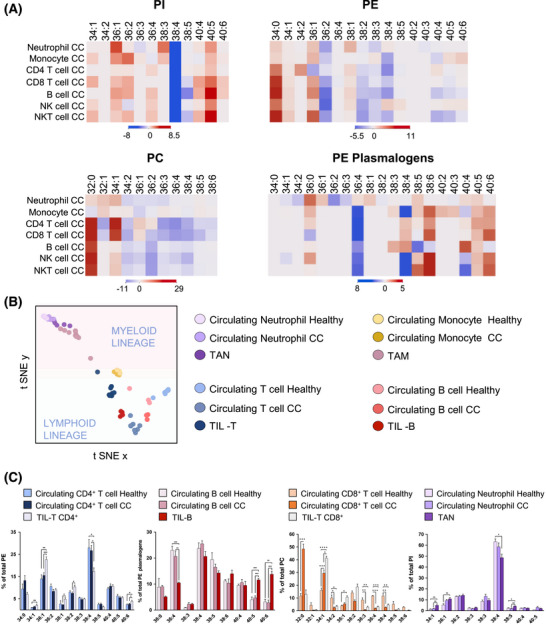
Impact of colon cancer (CC) on membrane lipid composition in immune cells. (A) Heatmaps illustrating alterations in the content of selected phospholipids in each immune cell type in patients with CC (CC, *n* = 5–8) compared to healthy donors (*n* = 5–7). (B) t‐SNE plot of circulating immune cell lipid fingerprints from healthy donors and patients with CC, and tumour‐infiltrating immune cells. Bar diagram depicting the differential phosphatidylethanolamine (PE) composition in the isolated tumour‐infiltrating immune populations. (C) Selected phospholipid profiles in circulating immune cells obtained from healthy donors and patients with CC (CC) and tumour‐infiltrating immune cells. Values represent the mean ± SEM, *n* = 5–7. Statistical significance was assessed by one‐way analysis of variance (ANOVA) followed by Bonferroni post‐test. *p < .05, **p < .01 and ***p < .001. For clarity, only species accounting for > 1% of the total lipid class were included.

Impressively, the activated phenotype and lipid remodelling described in circulating cells were reinforced and magnified in tumour‐infiltrated immune cells (both isolated and in situ) (Figure 3B). TIL‐T exhibited a severe depletion of AA‐species and an increase in MUFA‐species when compared to their circulating counterparts in both patients and healthy donors (Figure 3C and Figures S18 and S19). To assess a link with activation, CD25 expression was evaluated in TIL‐T. A substantial subset (14% of the total, *n* = 5) expressed CD25, confirming the presence of activated lymphocytes within the TME (Figure S20) and reinforcing that cell activation would account for the abovementioned specific lipid changes. In TIL‐B, docosahexaenoic acid‐containing PE‐plasmalogens increased compared to their circulating counterparts (Figure 3C and Figure S21). Additionally, consistent with the observations ex vivo, all immune populations displayed a sharp decline in PI38:4 compared to circulating populations (Figures S18–S24). Within the myeloid compartment, isolated M1‐ and M2‐like TAMs showed no significant differences when compared to each other (Figure S22). In patients, TAN presented similar trends to circulating neutrophils but, again, more marked (Figure S24). Finally, we mapped the spatial distribution of lipid species throughout CC biopsies, clearly identifying an MSI‐TIL cluster, namely the cluster best aligned with lymphocytic infiltrates, and obtained its lipid profile (Figure 4A–D and Figure S25). Comparison of the MSI‐TIL cluster with the isolated TIL‐T cell profile underscores the reliability of spatial lipidomic strategies in TME characterisation and validates the lipid profile consistency (*r* = 0.979) (Figure 4E,F)

**FIGURE 4:**
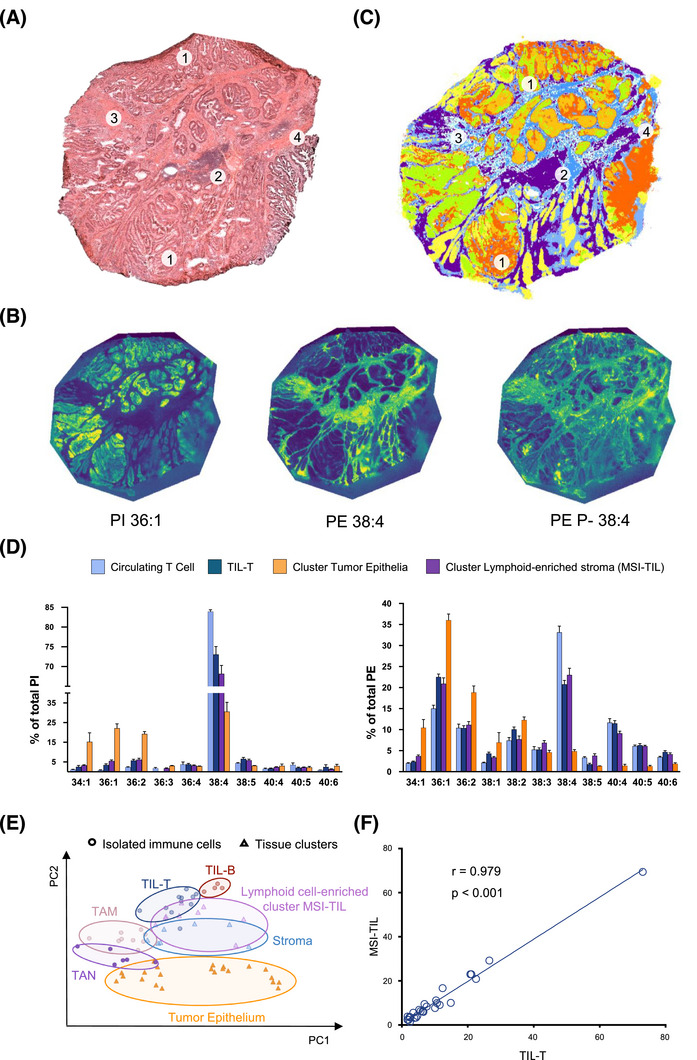
Matrix‐assisted laser desorption/ionisation mass spectrometry (MALDI‐MS) imaging analysis of colon cancer (CC) sections. (A) HE‐staining of surgical biopsies showing tumour epithelia (1), regions of high lymphocytic infiltration (2), stromal compartment (3), and muscular tissue (4). (B) MALDI‐MS imaging images of consecutive tissue sections recorded at 10 µm/pixel lateral resolution in negative ion mode and showing the spatial distribution of PI 36:1 (*m/z* 863.565), phosphatidylethanolamine (PE) 38:4 (*m/z* 766.539) and PE P‐38:4 (*m/z* 750.544) across tissue sections. (C) Lipidomic data segmentation of the consecutive tissue section in (a), performed using bisecting k‐means with correlation distance. (D) Bar diagrams comparing average PE and PI profiles at the species level of circulating T cells from patients with CC, TIL‐T, and clusters assigned to lymphocytic infiltrates and tumour epithelium. Values are expressed as percentages of the total content of each lipid class (*n* = 4). (E) PCA of lipid profiles correlating isolated tumour‐infiltrating immune populations with clusters generated during tumour section analysis from four patients. (F) Pearson's correlation analysis of lipid profiles between IMS‐TIL and TIL‐T populations, showing the correlation coefficient (r) and corresponding p‐values for lipid species. Statistical significance is denoted as *p < .05, **p < .01 and ***p < .001.

Overall, this study introduces a robust, low‐input lipidomic strategy with potential applications in early detection, therapeutic monitoring, and immune landscape characterisation in the TME.^10^ The analysis across ex vivo activation models, circulating, and tumour‐infiltrating immune cells revealed that both immune cell activation and infiltration, and the tumour presence elicit a profound impact on cell fatty acid profile. Although the limited sample size precludes broad clinical generalisations, our results provide proof‐of‐concept evidence. Furthermore, they underscore the potential of phospholipid profiling by MSI as a powerful tool for characterising the immune landscape in patients with CC, which could contribute to the refinement of patient stratification strategies, especially in the context of treatments like immunotherapy.

## AUTHOR CONTRIBUTIONS

Karim Pérez‐Romero curated data, conducted investigations and prepared the original draft of the manuscript. Cristina Huergo‐Baños and Lucía Martín‐Saíz participated in investigations and contributed to data curation. Albert Maimó‐Barceló also contributed to data curation and undertook investigation activities. Teresa Ximelis contributed to the investigation. Catalina Crespí, Marco A. Martínez, Paloma de la Torre and Myriam Fernández‐Isart were involved in the investigation. Daniel H. Lopez conceptualised the study. José Andrés Fernández contributed to the investigation and was involved in reviewing and editing the manuscript. Ramon M. Rodriguez participated in conceptualisation, investigation and also contributed to manuscript review and editing. Gwendolyn Barceló‐Coblijn was responsible for conceptualisation, secured funding for the study and participated in reviewing and editing the manuscript. All authors reviewed and approved the final version of the manuscript.

## CONFLICT OF INTEREST STATEMENT

All authors have read the journal's policy on disclosure of potential conflicts of interest. All authors disclose no financial or personal relationship with organisations that could potentially be perceived as influencing the research described.

## FUNDING INFORMATION

This study was supported in part by the Institute of Health Carlos III (PI19/00002, PI24/00313), the Basque Government (IT1491‐22) and the EC (European Regional Development Fund, ERDF). Karim Pérez‐Romero heldholds a predoctoral contract of the Health Institute Carlos III (FI20/00180) co‐funded by ESF (European Social Fund). Cristina Huergo‐Baños holds a contract funded by *Fundación Jesús Gangoiti Barrera and Grupo Multidisciplinar de Melanoma*. Albert Maimó‐Barceló held a predoctoral contract of the *Govern Balear (Direcció General d'Innovació i Recerca)* co‐funded by ESF (FPI/2160/2018). Currently, he holds a contract part of the grant (PTA2022‐021759‐I) funded by Ministerio de Ciencia, Innovación y Universidades (MCIU/AEI/10.13039/501100011033) and the ESF+ (European Social Fund+). Teresa Ximelis holds a contract funded by the Sustainable Tourism Tax Fund (ITS) of the Government of the Balearic Islands (ITS2023‐057). Lucía Martín‐Saíz held an FPI predoctoral contract funded by Ministerio de Ciencia, Innovación y Universidades (BES‐2016‐078721). Ramon M. Rodriguez holds a postdoctoral contract supported by the Scientific Foundation of the Spanish Association Against Cancer (INVES222995RODR).

## ETHICS STATEMENT

This study was approved by the Ethics Research Committee of the Balearic Islands (IB4568/21 PI), and all participants provided written informed consent.

## Supporting information



Supporting Information

Supporting Information

Supporting Information

Supporting Information

## Data Availability

This study includes no data deposited in external repositories. The data that support the findings of this study are available from Gwendolyn Barceló‐Coblijn upon reasonable request.
