# Supplementary material for: Advancing immune profiling in colon cancer through enhanced lipidomics of low‐input samples
Source: Clin Transl Med. 2025 Jul 11;15(7):e70399. doi: 10.1002/ctm2.70399 (PMC12246954; doi:10.1002/ctm2.70399)
Supplement: Supplementary file 1 — Supporting Information [file CTM2-15-e70399-s002.pdf]

Supplementary Materials and Methods

Advancing immune profiling in colon cancer through enhanced lipidomics of low-input samples

Short title: Colon cancer impacts on immune cells lipidome

Karim Pérez-Romero[1,2], Cristina Huergo Baños[3], Albert Maimó-Barceló[1,2], Lucía Martín-Saíz[3], Teresa Ximelis[1,2], Catalina Crespí-Bestard[1,2], Marco A. Martínez[1,2,4], Paloma de la Torre[1,2,5], Myriam Fernández-Isart[1,2,6], Daniel H. Lopez[1,2], José Andrés Fernández[3], Ramon M. Rodriguez[1,2,*], Gwendolyn Barceló-Coblijn[1,2,*]

[1] Institut d'Investigació Sanitària Illes Balears (IdISBa) – Health Research Institute of the Balearic Islands, Ctra. Valldemossa 79, Module G, Floor -1, E-07120, Palma, Balearic Islands, Spain;

[2] Research Unit of the Hospital Universitari Son Espases, 07120 Palma, Balearic Islands, Spain;

[3] Department of Physical Chemistry, Fac. of Science and Technology, University of the Basque Country (UPV/EHU), Barrio Sarriena s/n, 48940 Leioa, Spain.

[4] Department of Pathology Anatomy, Hospital Universitari Son Espases, Ctra. Valldemossa 79, 07120 Palma, Balearic Islands, Spain;

[5] Department of Gastroenterology, Hospital Universitari Son Espases, Ctra. Valldemossa 79, 07120 Palma, Balearic Islands, Spain;

[6] Department of Gastroenterology and General Surgery, Hospital Universitari Son Espases, Ctra. Valldemossa 79, 07120 Palma, Balearic Islands, Spain;

*Contact information for corresponding author:

Gwendolyn Barceló-Coblijn, PhD

Lipids in Human Pathology, Institut d'Investigació Sanitària Illes Balears (IdISBa), Health Research Institute of the Balearic Islands, Carretera de Valldemossa 79, Module G, Floor -1, E-07120, Palma, Balearic Islands, Spain, email: gwendolyn.barcelo@ssib.es

Ramon Maria Rodriguez, PhD

Lipids in Human Pathology, Institut d'Investigació Sanitària Illes Balears (IdISBa), Health Research Institute of the Balearic Islands, Carretera de Valldemossa 79, Module G, Floor -1, E-07120, Palma, Balearic Islands, Spain, email: ramonmaria.rodriguez@ssib.es

Supplementary Figures Index:

Figure S1. Gating strategy for the isolation by FACS of circulating immune cells.
Figure S2. Lipid fingerprint of 7 circulating immune cell populations obtained from healthy donors.
Figure S3. In vitro activation of T lymphocytes and differentiation of monocytes isolated from human peripheral blood.
Figure S4. Lipid signature of ex-vivo activated $CD4^+$ and $CD8^+$ naive T Cells.
Figure S5. Lipid signature of ex-vivo activated naive B Cells.
Figure S6. Lipid signature of ex-vivo activated neutrophils and monocytes.
Figure S7. Lipid signature of ex vivo monocyte-derived macrophages (GM-CSF and M-CSF).
Figure S8. Transcriptomic analysis of monocyte-derived macrophages and CD4+ and CD8+ T cells activated ex vivo.
Figure S9. Impact of colon cancer on membrane lipid composition in circulating immune cells
Figure S10. Lipid signature of circulating monocytes.
Figure S11. Lipid signature of circulating $CD4^+$ T Cells.
Figure S12. Lipid signature of circulating $CD8^+$ T Cells.
Figure S13. Lipid signature of circulating B Cells.
Figure S14. Lipid signature of circulating NK Cell.
Figure S15. Lipid signature of circulating neutrophil.
Figure S16. Lipid signature of circulating NK T Cell.
Figure S17. Reduction in AA- and LA-lipids in circulating immune cells from patients with colon cancer.
Figure S18. Lipid signature of circulating $CD4^+$ T Cells from healthy donors and CC patients and tumor-infiltrating $CD4^+$ lymphocytes.
Figure S19. Lipid signature of circulating $CD8^+$ T Cells from healthy donors and CC patients and tumor-infiltrating $CD8^+$ lymphocytes.
Figure S20. Immunophenotyping panel for the identification and isolation of TIL.
Figure S21. Lipid signature of circulating B Cells from healthy donors and CC patients and tumor-infiltrating B Cells
Figure S22. Lipid signature of M1 and M2-like tumor-associated macrophages (TAM).
Figure S23. Lipid signature of circulating monocytes from healthy donors, CC patients, and M1 and M2-like tumor-associated macrophages (TAM).
Figure S24. Lipid signature of circulating neutrophils from healthy donors and CC patients and tumor-associated neutrophils (TAN).
Figure S25. Immunofluorescence analysis of T cell infiltration in relation to histological and MSI lipidomic features.
Table S3. Clinical and histopathological characteristics of patients.

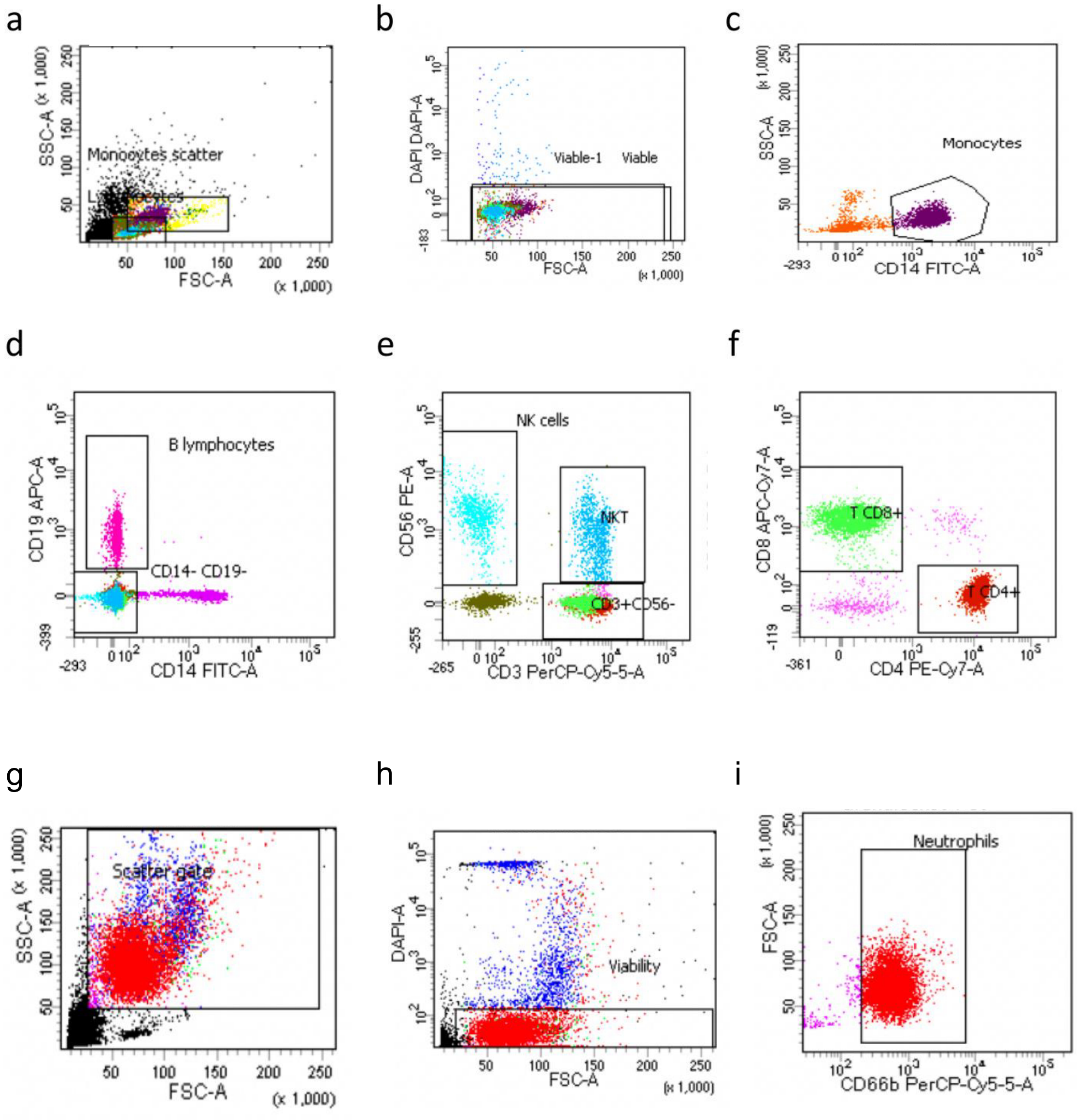

Figure S1. Gating strategy for the isolation by FACS of circulating immune cells. Initial identification (a) is based on cell size (FSC-A) and granularity/complexity (SSC-A). (b) Cell viability is determined by the absence of DAPI staining. Immunophenotyping is performed by analyzing the fluorescence of conjugated antibodies specific to the immune cell types of interest: monocytes (c), B cells (d), NK cells (e), NKT cells (e), and CD4+ and CD8+ T cells. (g) Granulocytes are identified by their high granularity/complexity (SSC-A). (h) Among the cells with high SSC-A values, viable events were selected, and within this population, (i) neutrophils were identified based on CD66b+ expression.

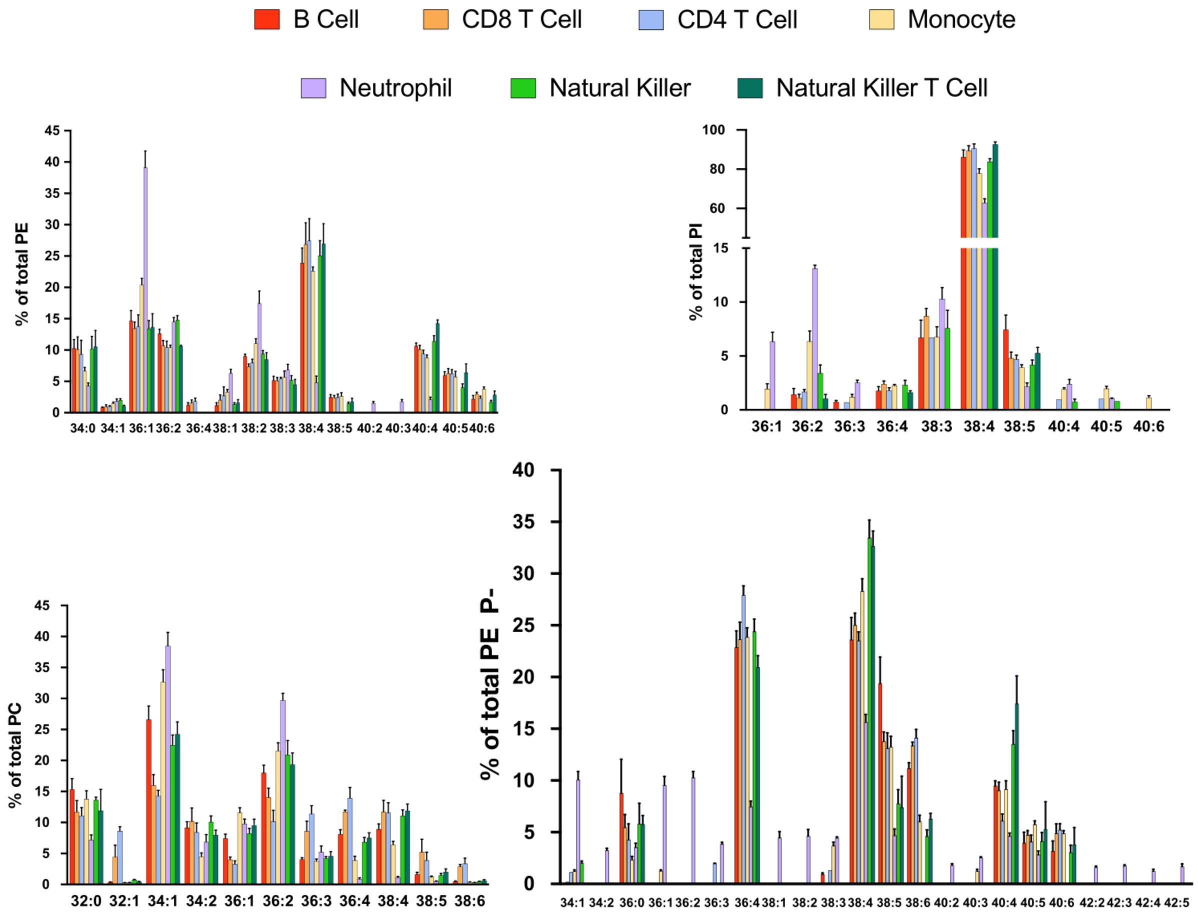

Figure S2. Lipid fingerprint of 7 circulating immune cell populations obtained from healthy donors. Data is represented considering the content in % of each of the lipid species that make up the main lipid classes detected on negative ion mode, phosphatidylethanolamine (PE), phosphatidylinositol (PI), phosphatidylethanolamine plasmalogens (PE plasmalogens), and phosphatidylcholine (PC). Only species accounting for > 1% of the total membrane lipid class are included in the graph.

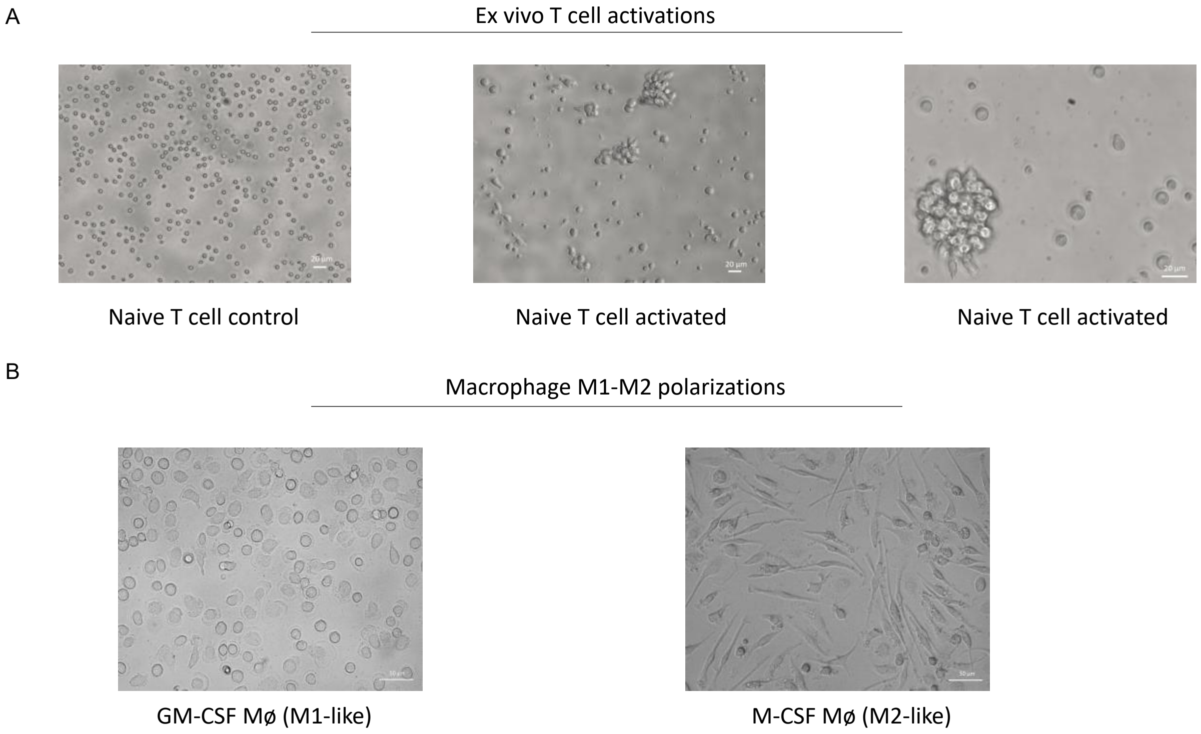

Figure S3. In vitro activation of T lymphocytes and differentiation of monocytes isolated from human peripheral blood. (**A**) Representative images of naïve T cells before (left, unstimulated) and after stimulation (right), showing characteristic clonal expansion associated with activation. (**B**) Representative images of monocytes differentiated for 7 days in the presence of GM-CSF (left) or M-CSF (right), illustrating the distinct adherent morphologies of resulting macrophage subtypes.

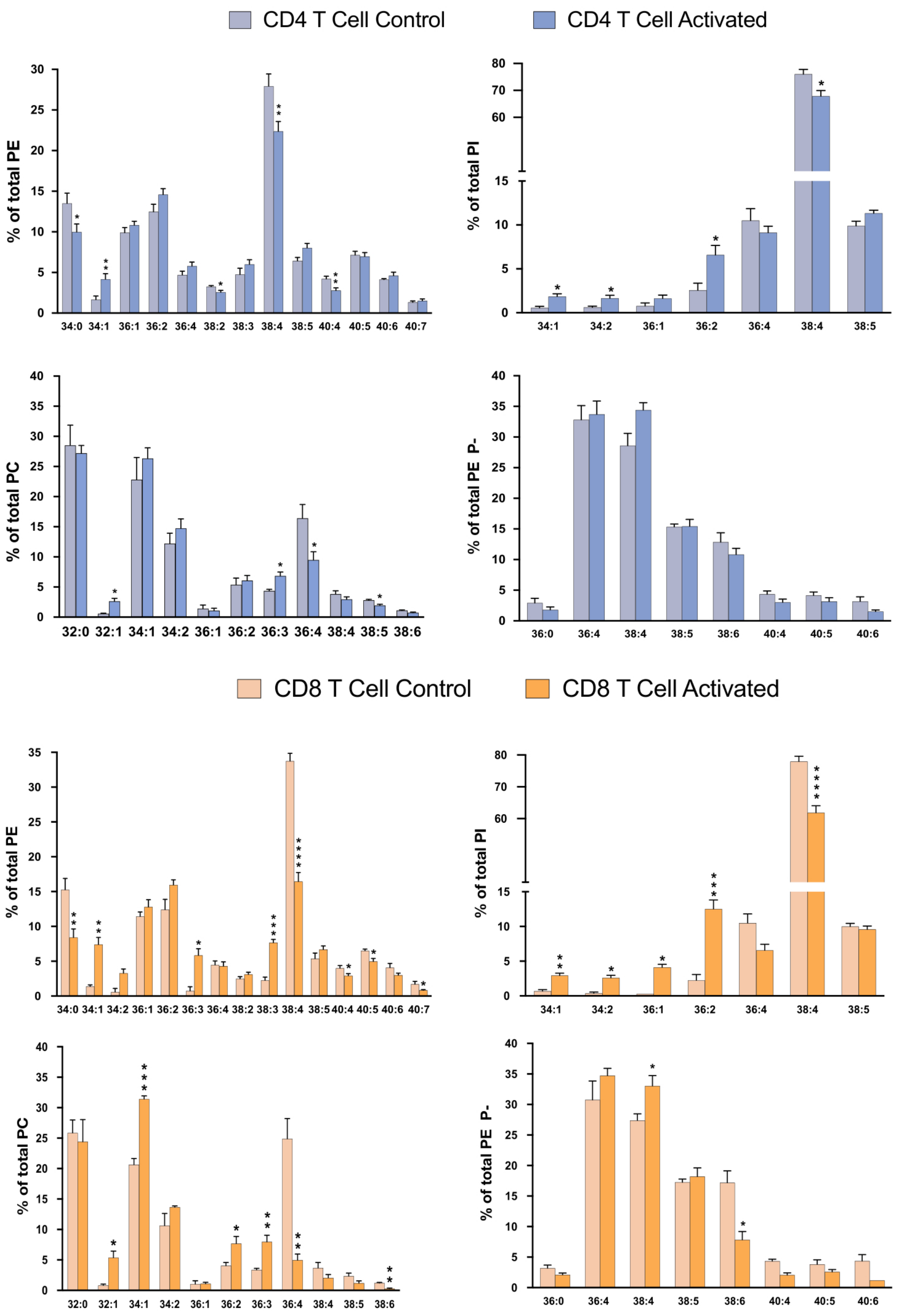

Figure S4. Lipid signature of ex-vivo activated CD4⁺ and CD8⁺ naive T Cells. Bar graphs. Bar diagrams showing the comparison of lipid species content within each lipid class between CD4⁺ naive (light blue) and CD8⁺ naive (light orange); and activated CD4⁺ (dark blue) and activated CD8⁺ (dark orange). Values are represented as % total phospholipids of each lipid species within the main lipid classes detected in negative- (PE, PI and PE P-) and positive-ion (PC) modes. Values represent mean ± SEM, n = 8-10 PE, PI and PE P-, n=3-6 for PC. Statistical significance was assessed using an unpaired Student's *t*-test, comparing activated to control cells. The asterisk (*) indicates a significant difference between activated CD4⁺ naive T Cells and cultured unstimulated (control) naive CD4⁺ T cells. * $P < .05$, ** $P < .01$. Only species accounting for > 1% of the total membrane lipid class are included in the graph.

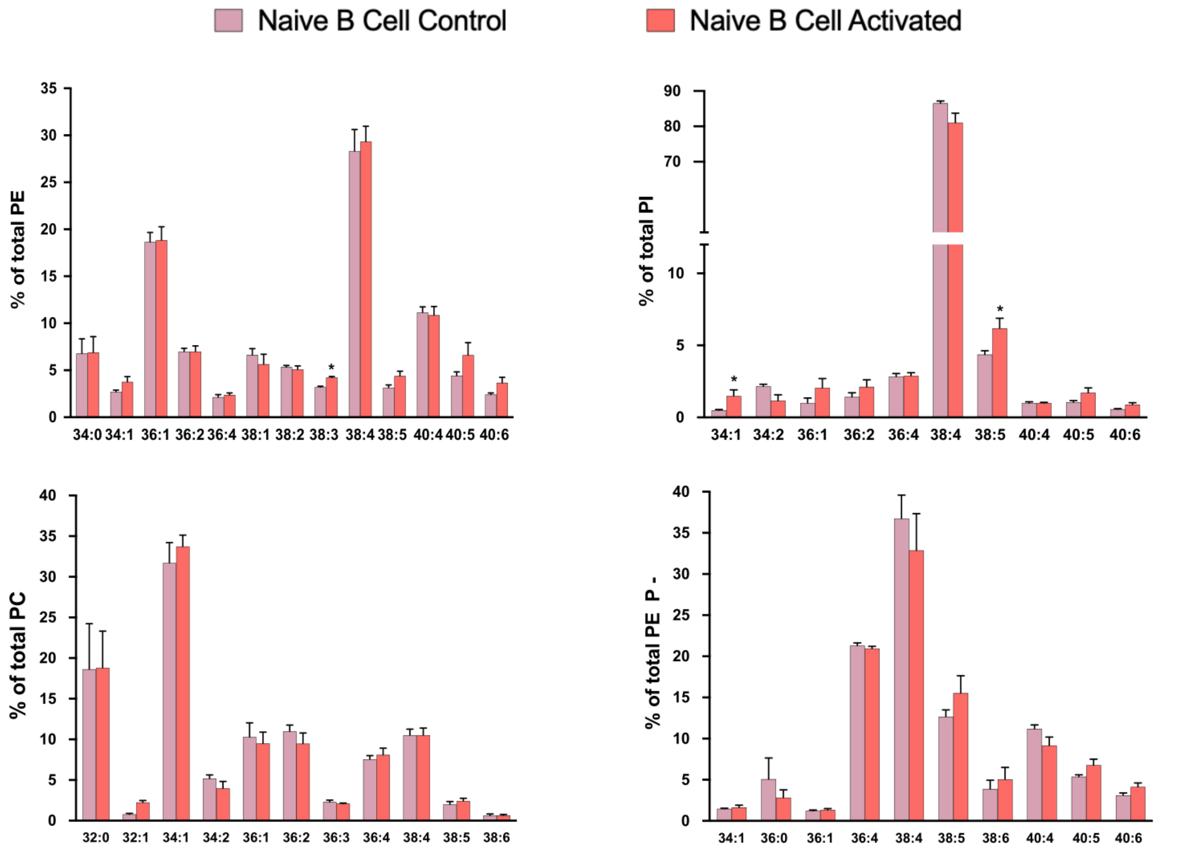

Figure S5. Lipid signature of ex-vivo activated naive B Cells. Lipid species content within each lipid class**.** Values are represented as % for each lipid species within the main lipid classes detected on both negative and positive ion modes. Represent mean ± SEM, n = 5. Statistical significance was assessed using an unpaired Student's *t*-test, comparing activated to control cells. The asterisk (*) indicates a significant difference between activated naive B Cells and cultured unstimulated (control) naive B cells. * P < .05, ** P < .01. Only species accounting for > 1% of the total membrane lipid class are included in the graph.

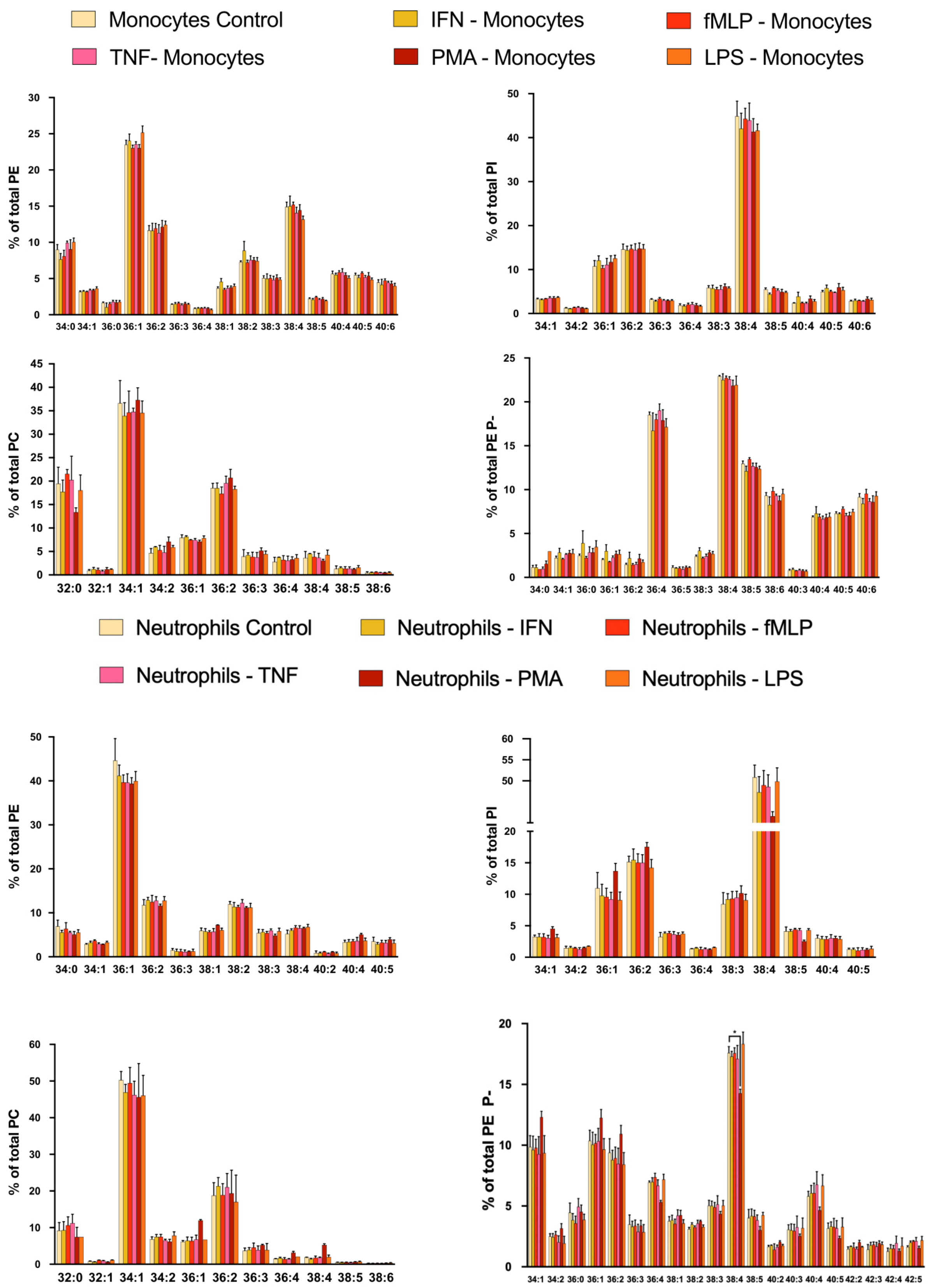

Figure S6. Lipid signature of ex-vivo activated neutrophils and monocytes. Values are represented as % for each lipid species within the main lipid classes detected on negative ion mode. Represent mean ± SEM, n = 4-5. Statistical significance was assessed by one-way ANOVA followed by Bonferroni post-test. * P < .05. Only species accounting for > 1% of the total membrane lipid class are included in the graph.

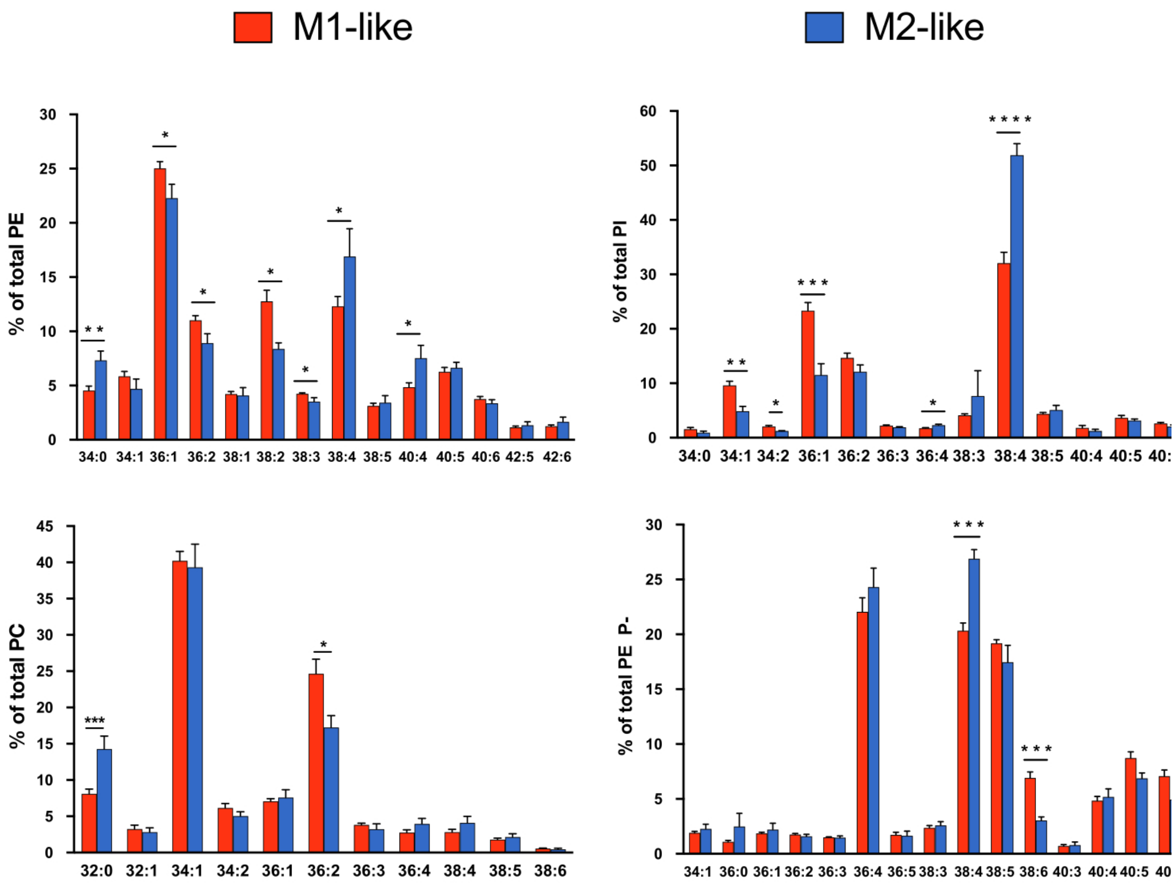

Figure S7. Lipid signature of ex vivo monocyte-derived macrophages (GM-CSF and M-CSF). Bar diagrams showing the content within each lipid class in M1- and M2-like macrophages. Values are represented as % for each lipid species within each of the main lipid classes detected on negative ion mode. Represent mean ± SEM, n = 6-17. Statistical significance was assessed using an unpaired Student's *t*-test, comparing GM-CSG to M-CSF-treated monocyte-derived macrophages. The asterisk (*) indicates a significant difference between GM-CSG to M-CSF-treated monocyte-derived macrophages. * P < .05, ** P < .01, *** P <.001. Only species accounting for > 1% of the total membrane lipid class are included in the graph.

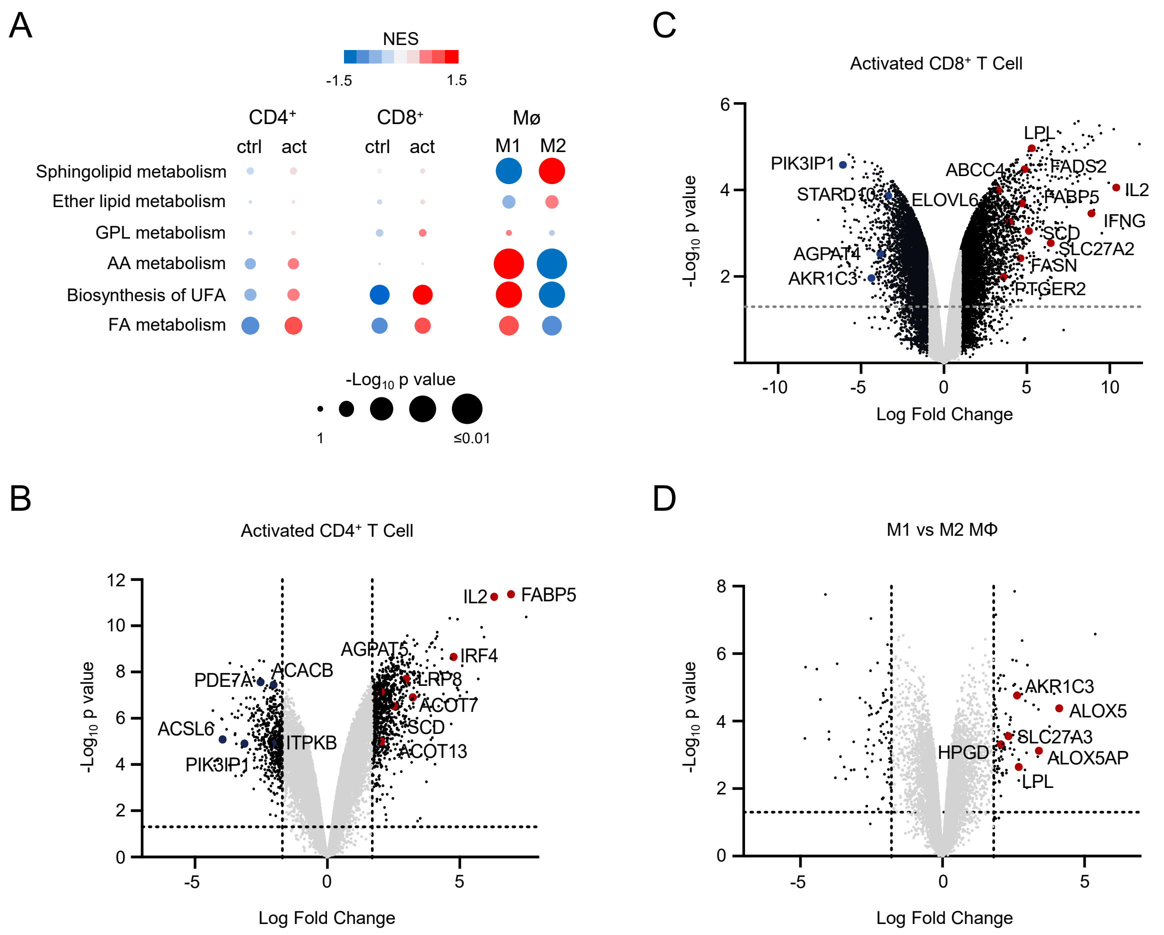

Figure S8. Transcriptomic analysis of monocyte-derived macrophages and CD4⁺ and CD8⁺ T cells activated ex vivo. (a) GSEA showing normalized enrichment score (NES) of gene sets associated with lipid metabolism. (b-d) Volcano plots illustrating differentially expressed genes in activated CD4+ and CD8+ T cells and between M1 and M2 macrophages.

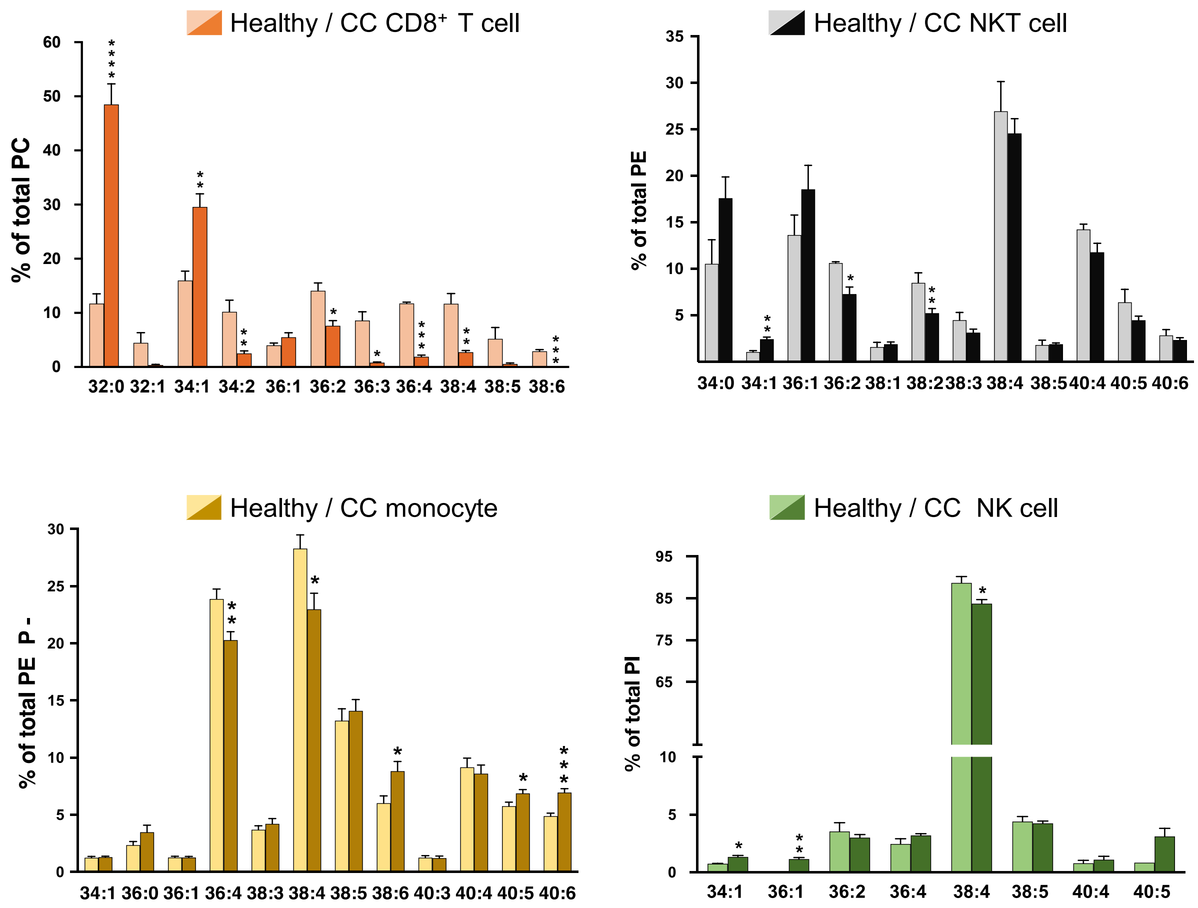

Figure S9. Impact of colon cancer on membrane lipid composition in circulating immune cells. Alterations in the content of PC, PE, PE plasmalogen, and PI in each immune cell type in patients with colon cancer (CC, n = 5-8) compared to healthy donors (n = 5-7). In the bar graphs, values represent mean ± SEM. Statistical significance was assessed using an unpaired Student's t-test. * P <.05, ** P <.01, *** P <.001.

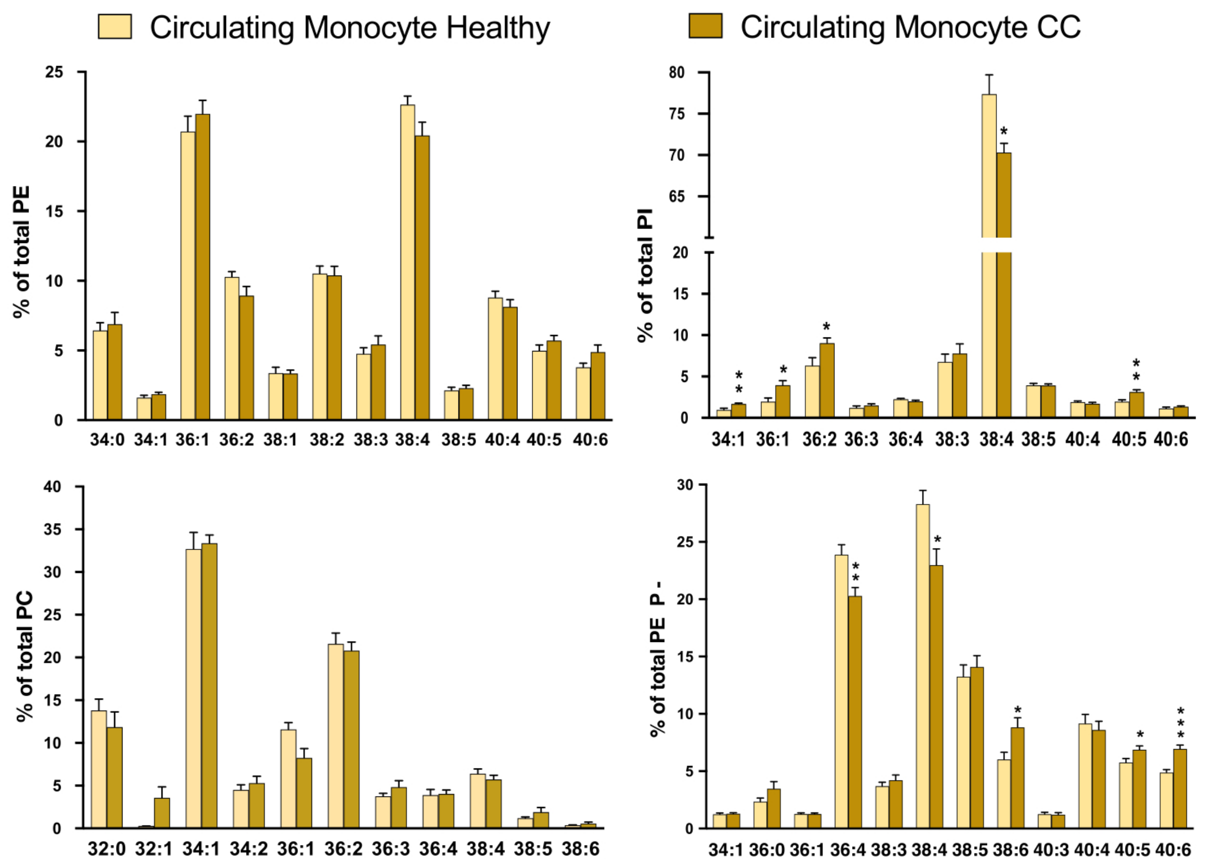

Figure S10. Lipid signature of circulating monocytes. Bar diagrams showing the content within each lipid class in circulating monocytes obtained from healthy donors and CC patients. Values are represented as % for each lipid species within each of the main lipid classes detected on negative ion mode. Represent mean ± SEM, n = 5-7. Statistical significance was assessed using an unpaired Student's *t*-test, comparing the membrane lipid profiles of monocytes of healthy donors and CC patients. The asterisk (*) indicates a significant difference between monocytes of healthy donors and CC patients. * P < .05, ** P < .01, *** P < .001. Only species accounting for > 1% of the total membrane lipid class are included in the graph.

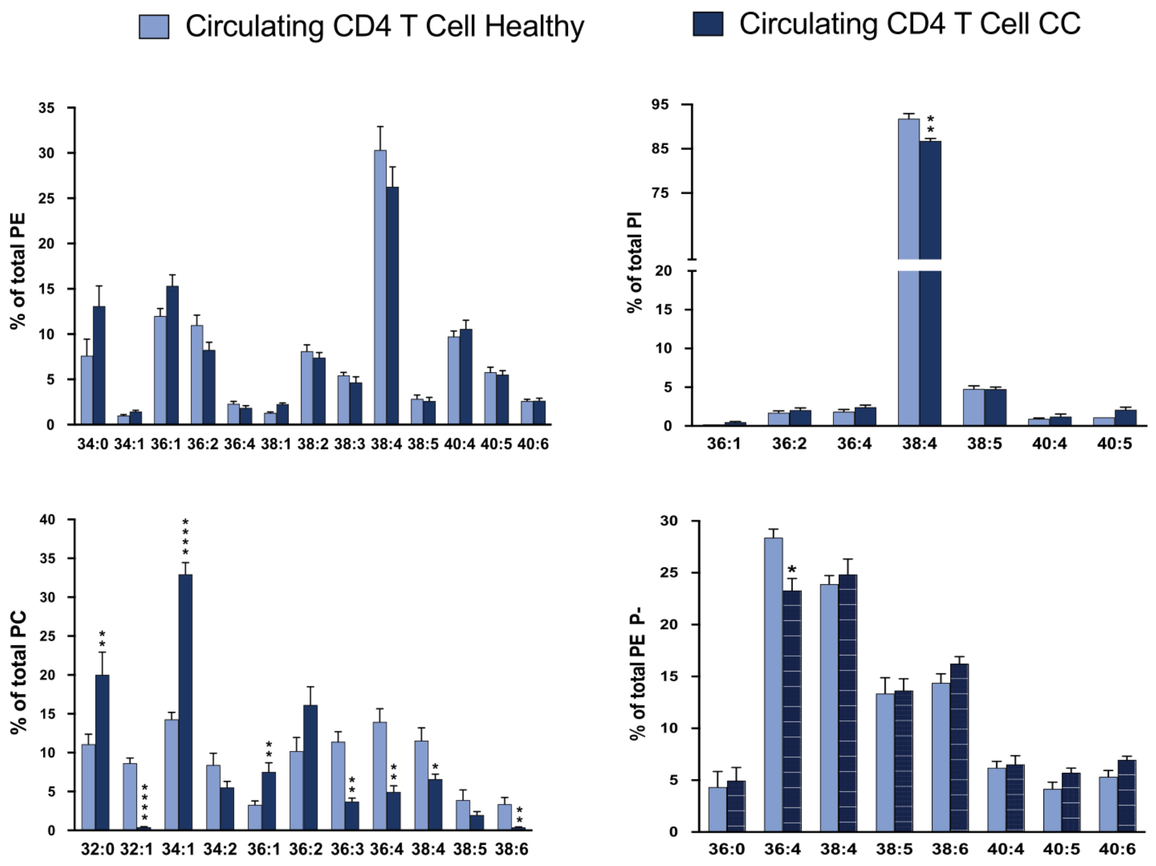

Figure S11. Lipid signature circulating CD4⁺ T Cells. Bar diagrams showing the content within each lipid class in circulating CD4⁺ T Cells obtained from healthy donors and CC patients. Values are represented as % for each lipid species within each of the main lipid classes detected on negative ion mode. Represent mean $\pm$ SEM, n = 5-7. Statistical significance was assessed using an unpaired Student's *t*-test, comparing the membrane lipid profiles of CD4+ T cells of healthy donors and CC patients. The asterisk (*) indicates a significant difference between CD4+ T cells of healthy donors and CC patients. * P < .05, ** P < .01, *** P <.001. Only species accounting for > 1% of the total membrane lipid class are included in the graph.

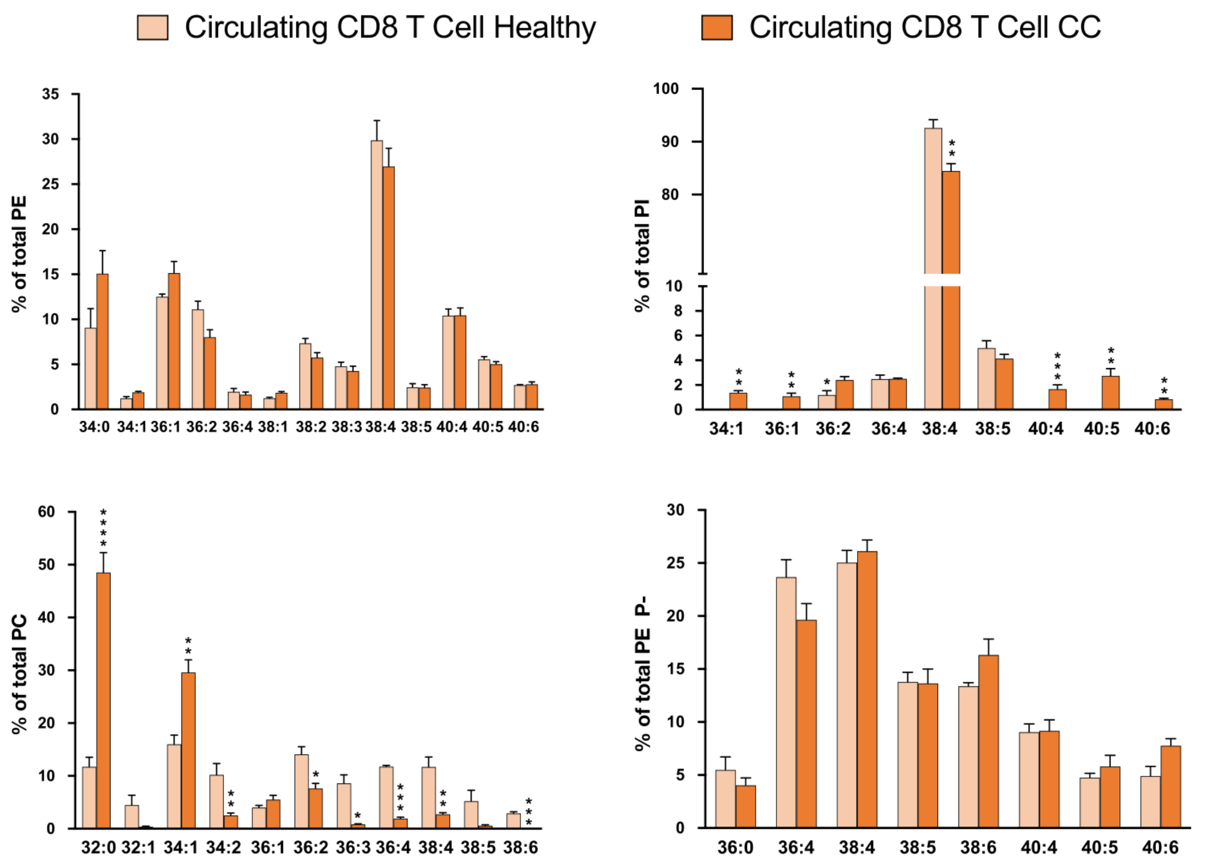

Figure S12. Lipid signature circulating CD8^{+} T Cells. Bar diagrams showing the content within each lipid class in circulating CD8^{+} T Cells obtained from healthy donors and CC patients. Values are represented as % for each lipid species within each of the main lipid classes detected on negative ion mode. Represent mean $\pm$ SEM, n = 5-7. Statistical significance was assessed using an unpaired Student's *t*-test, comparing the membrane lipid profiles of CD8^{+} T cells of healthy donors and CC patients. The asterisk (*) indicates a significant difference between CD8^{+} T cells of healthy donors and CC patients. * P < .05, ** P < .01, *** P <.001. Only species accounting for > 1% of the total membrane lipid class are included in the graph.

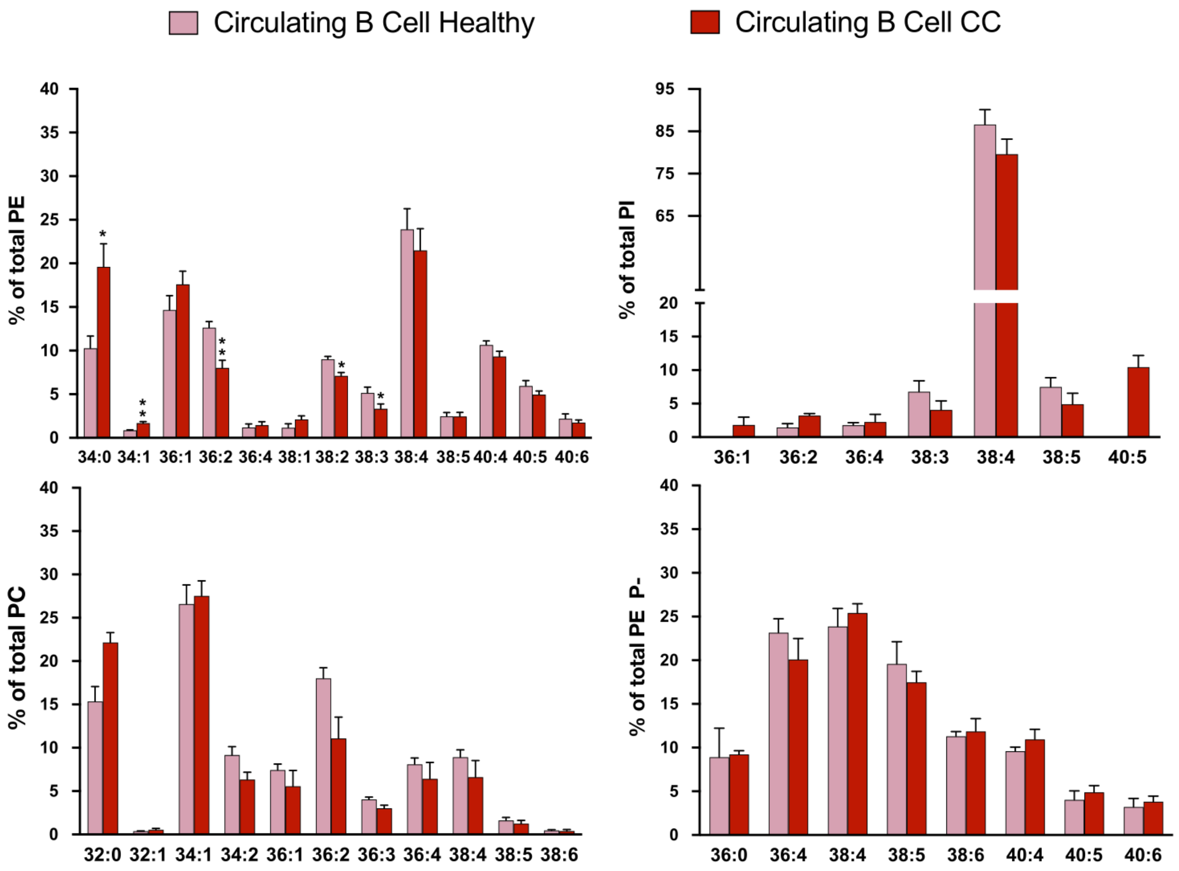

Figure S13. Lipid signature circulating B Cells. Bar diagrams showing the content within each lipid class in circulating B Cells obtained from healthy donors and CC patients. Values are represented as % for each lipid species within each of the main lipid classes detected on negative ion mode. Represent mean ± SEM, n = 5-7. Statistical significance was assessed using an unpaired Student's *t*-test, comparing the membrane lipid profiles of B cells of healthy donors and CC patients. The asterisk (*) indicates a significant difference between B cells of healthy donors and CC patients. * P < .05, ** P < .01, *** P <.001. Only species accounting for > 1% of the total membrane lipid class are included in the graph.

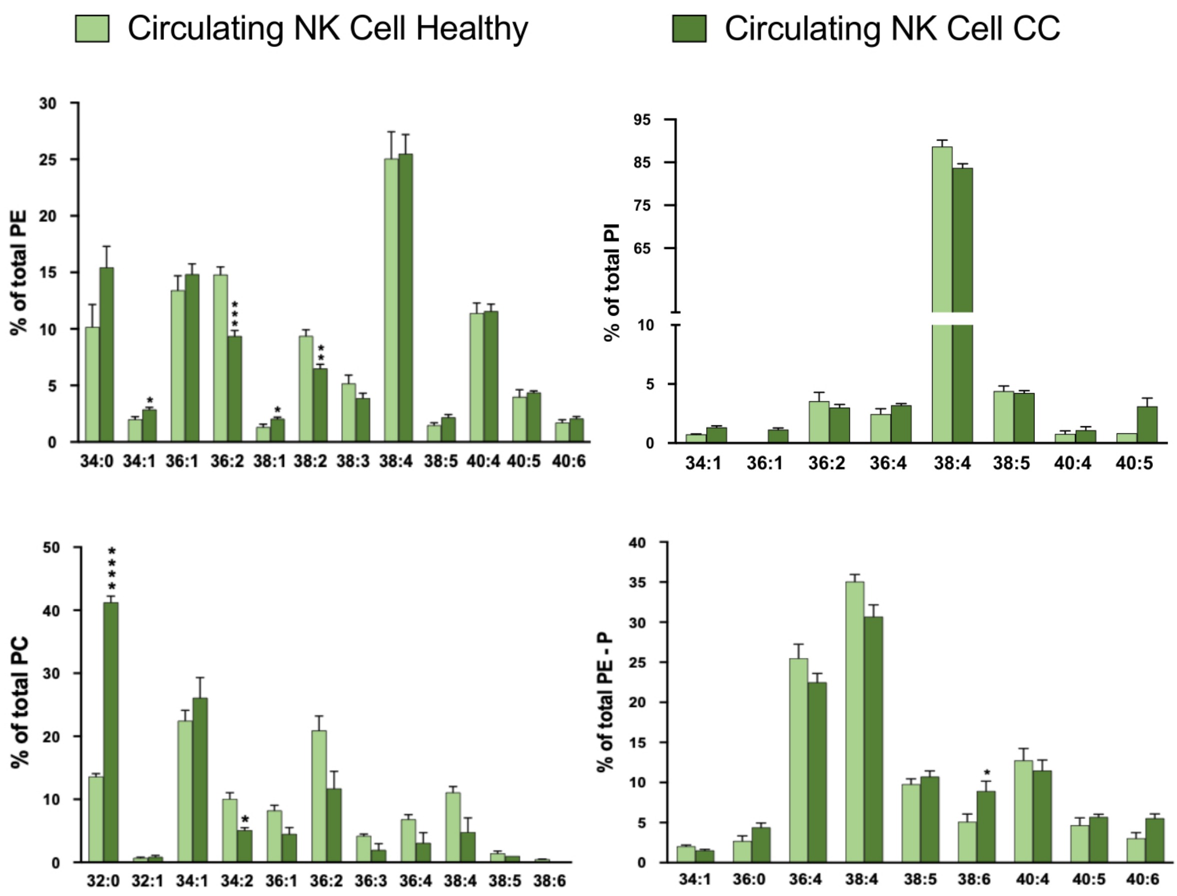

Figure S14. Lipid signature of circulating NK Cell. Bar diagrams showing the content within each lipid class in circulating NK Cell obtained from healthy donors and CC patients. Values are represented as % for each lipid species within each of the main lipid classes detected on negative ion mode. Represent mean $\pm$ SEM, n = 5-7. Statistical significance was assessed using an unpaired Student's *t*-test, comparing the membrane lipid profiles of NK Cell of healthy donors and CC patients. The asterisk (*) indicates a significant difference between NK Cell of healthy donors and CC patients. * P < .05, ** P < .01, *** P < .001. Only species accounting for > 1% of the total membrane lipid class are included in the graph.

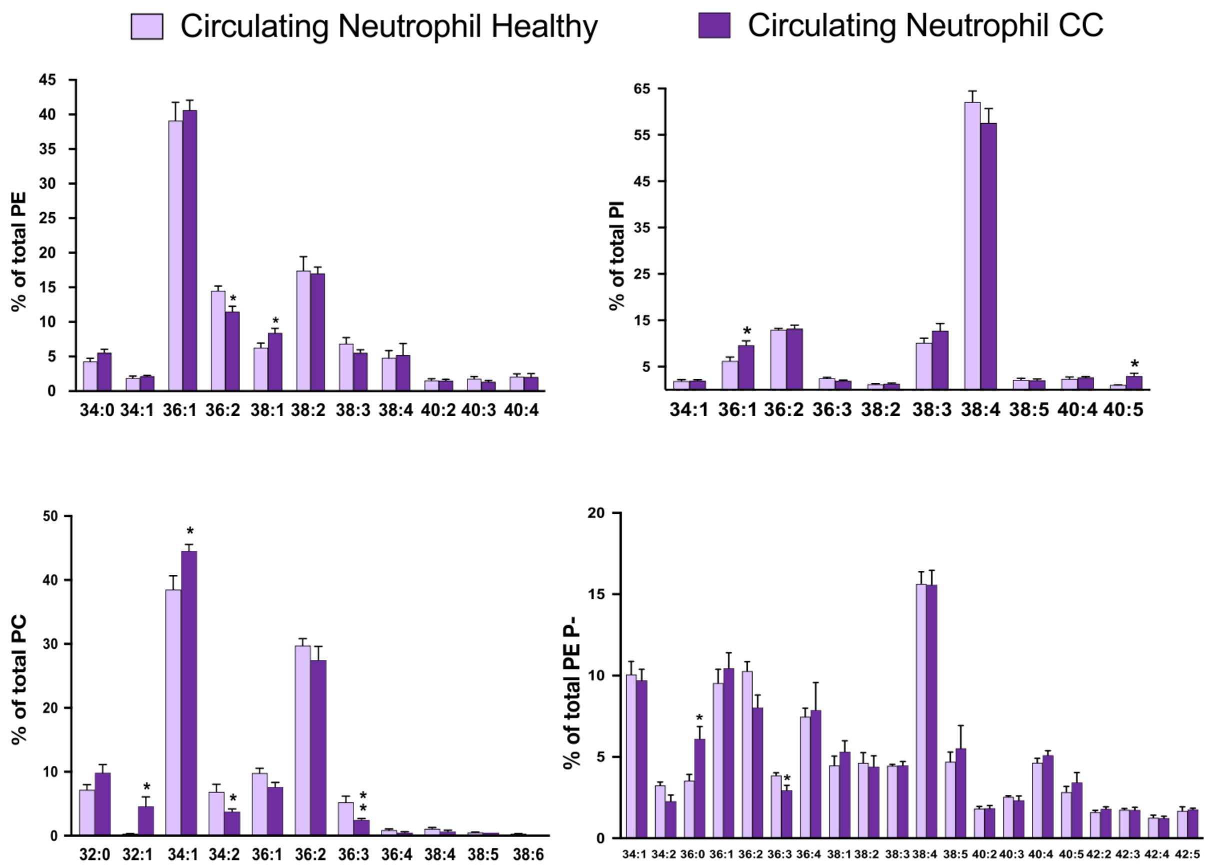

Figure S15. Lipid signature of circulating neutrophils. Bar diagrams showing the content within each lipid class in circulating neutrophils obtained from healthy donors and CC patients. Values are represented as % for each lipid species within each of the main lipid classes detected on negative ion mode. Represent mean ± SEM, n = 5-7. Statistical significance was assessed using an unpaired Student's *t*-test, comparing the membrane lipid profiles of neutrophils of healthy donors and CC patients. The asterisk (*) indicates a significant difference between neutrophils of healthy donors and CC patients. * P < .05, ** P < .01, *** p < .001. Only species accounting for > 1% of the total membrane lipid class are included in the graph.

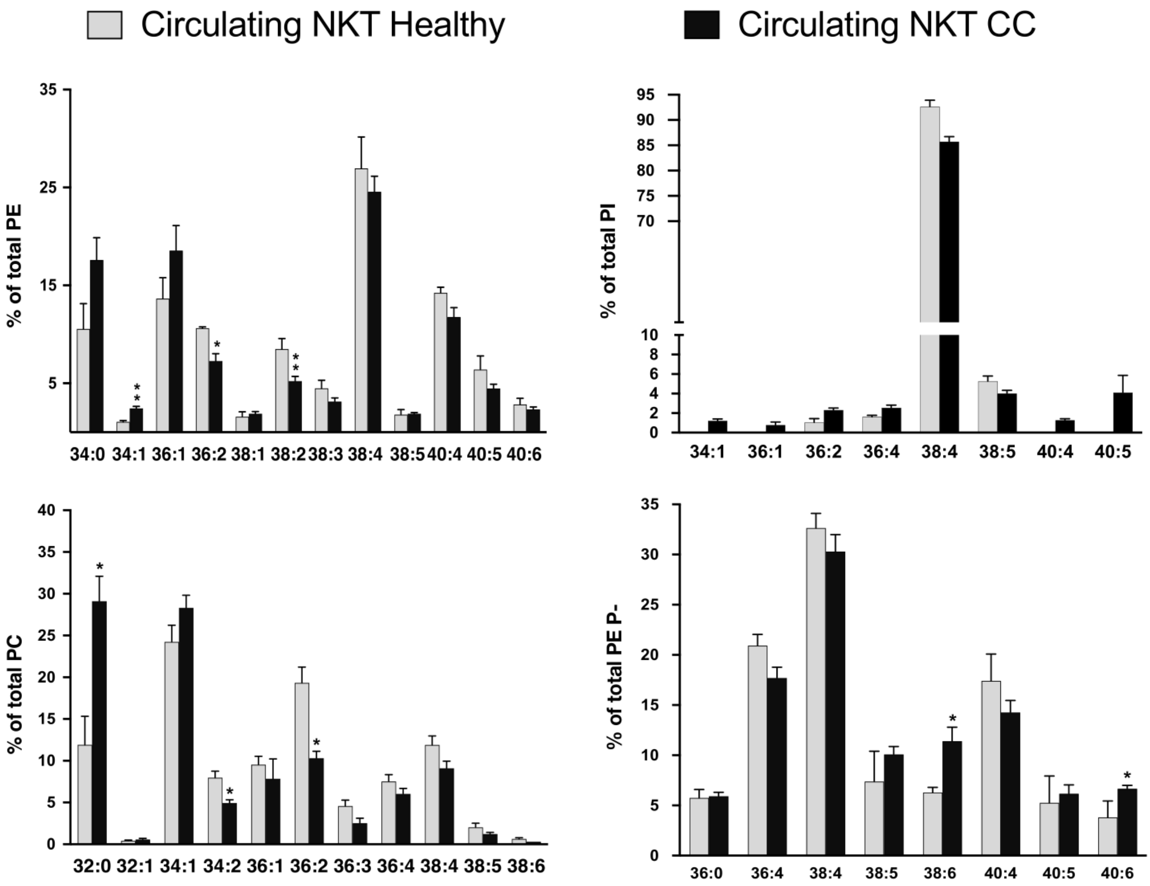

Figure S16. Lipid signature of circulating NKT Cells. Bar diagrams showing the content within each lipid class in circulating NKT Cells obtained from healthy donors and CC patients. Values are represented as % for each lipid species within each of the main lipid classes detected on negative ion mode. Represent mean ± SEM, n = 5-7. Statistical significance was assessed using an unpaired Student's *t*-test, comparing the membrane lipid profiles of NKT Cells of healthy donors and CC patients. The asterisk (*) indicates a significant difference between NKT Cells of healthy donors and CC patients. * P < .05, ** P < .01, *** P < .001. Only species accounting for > 1% of the total membrane lipid class are included in the graph.

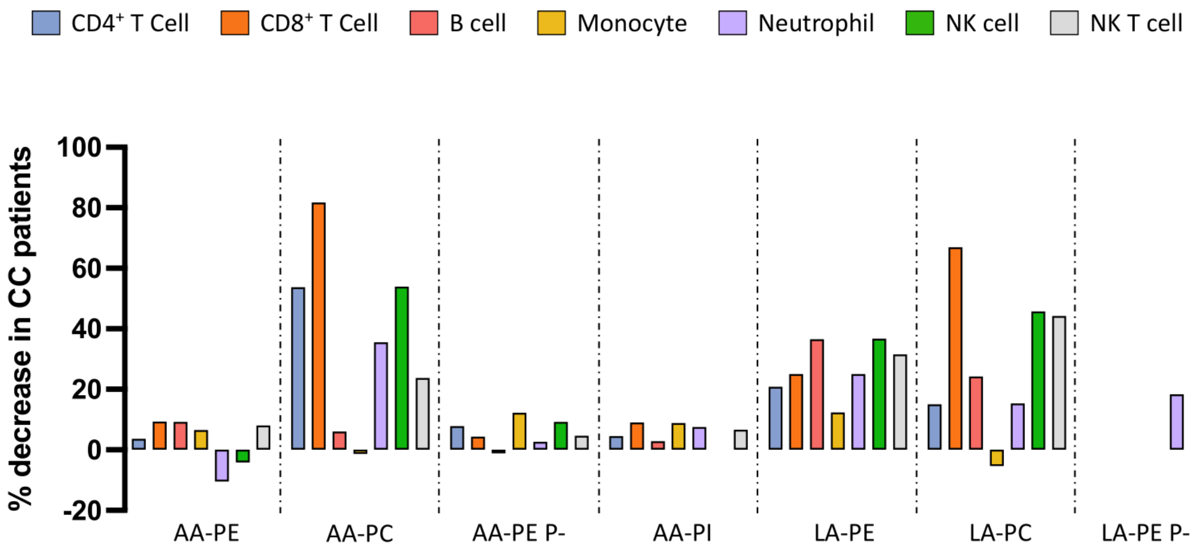

Figure S17. Reduction in AA- and LA-lipids in circulating immune cells from patients with colon cancer: Bar graphs depicting the percentage reduction in AA and LA-containing species across major lipid classes in circulating immune cell populations from patients with colon cancer compared to healthy donors. Data reflect the percentage decrease in AA and LA species relative to their content within each lipid class compared to the same immune cell population in healthy donors. The data highlight specific compositional changes across immune cell types and lipid classes.

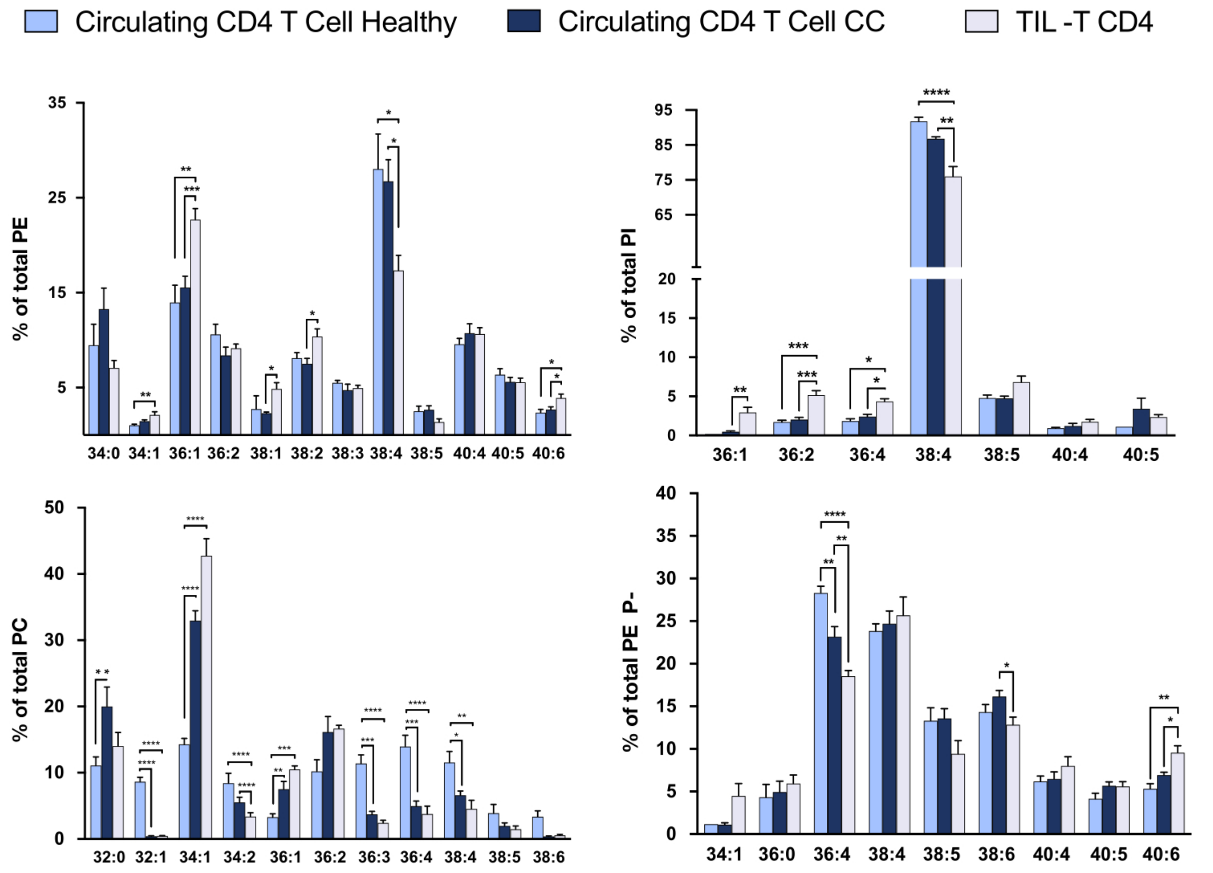

Figure S18. Lipid signature of circulating CD4+ T Cells from healthy donors and CC patients and tumor-infiltrating CD4+ lymphocytes. Bar diagrams showing the content within each lipid class in circulating CD4+T cells obtained from healthy donors and CC patients and CD4+ TIL. Values are represented as % for each lipid species within each of the main lipid classes detected on negative ion mode. Represent mean + SEM, n = 5-7. Statistical significance was assessed by one-way ANOVA followed by Bonferroni post-test. * P < .05, ** P < .01, *** P < .001. Only species accounting for > 1% of the total membrane lipid class are included in the graph.

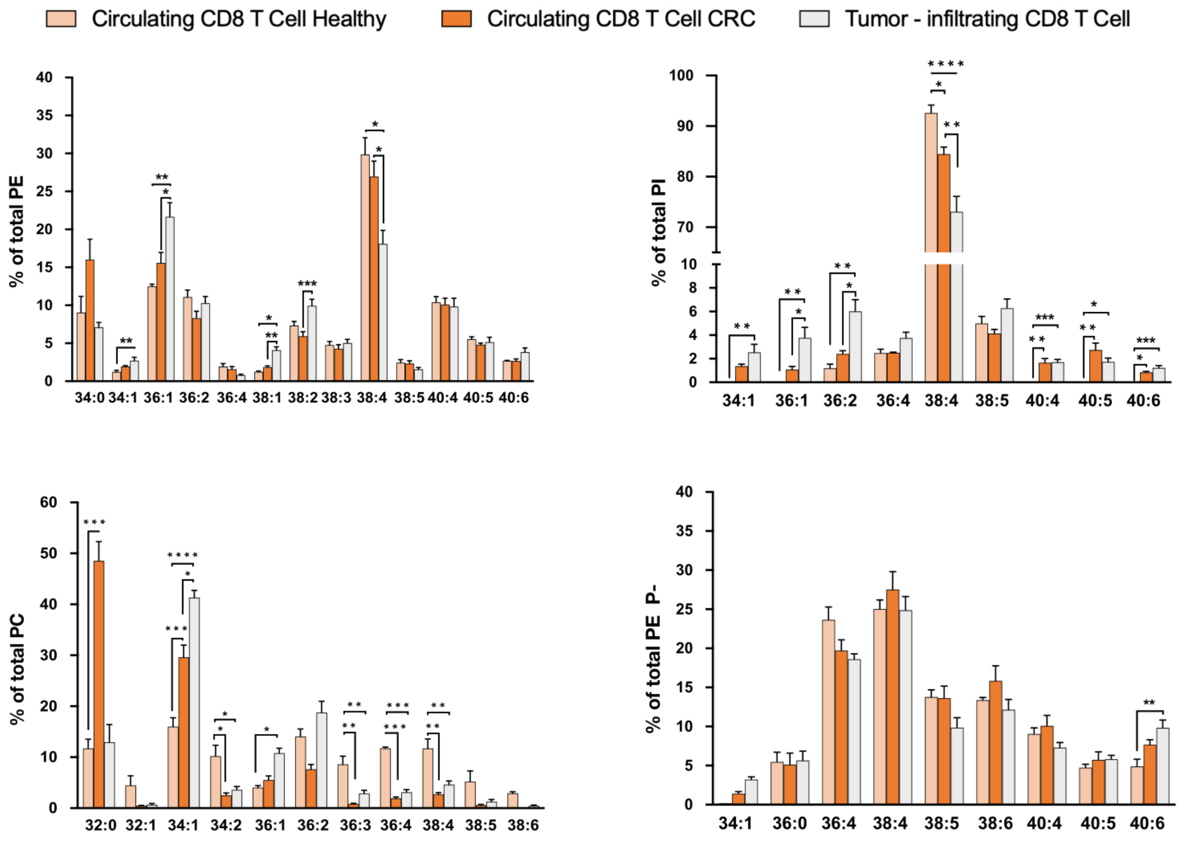

Figure S19. Lipid signature of circulating CD8⁺ T Cells from healthy donors and CC patients and tumor-infiltrating CD8⁺ lymphocytes. Bar diagrams showing the content within each lipid class in circulating CD8⁺T cells obtained from healthy donors and CC patients and CD8⁺ TIL. Values are represented as % for each lipid species within each of the main lipid classes detected on negative ion mode. Represent mean ± SEM, n = 5-7. Statistical significance was assessed by one-way ANOVA followed by Bonferroni post-test. * $P < .05$, ** $P < .01$, *** $P < .001$. Only species accounting for > 1% of the total membrane lipid class are included in the graph.

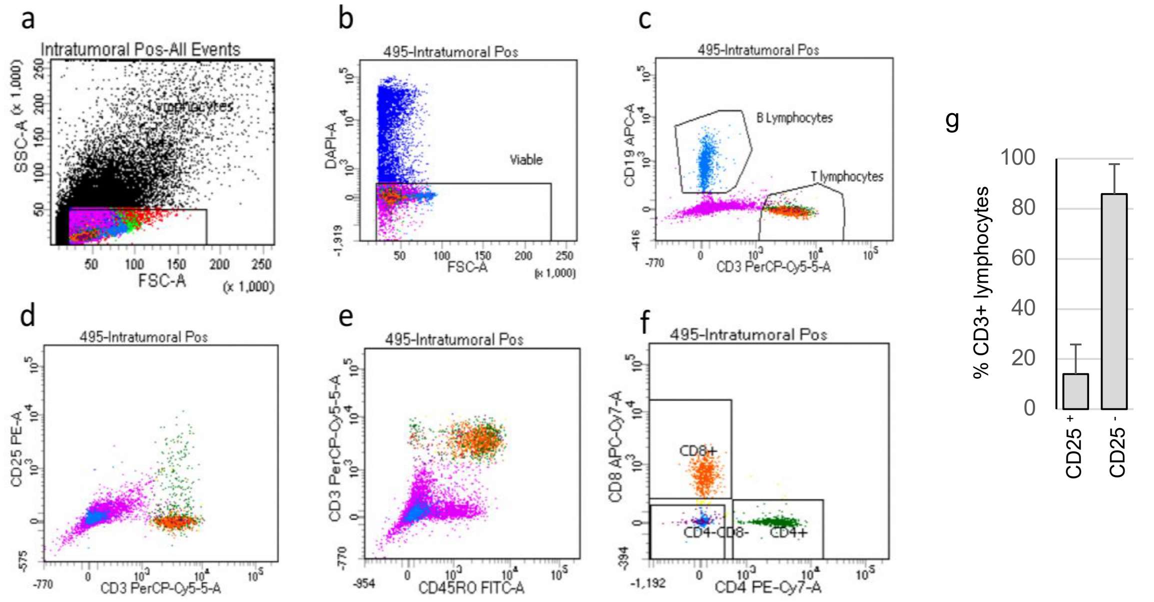

Figure S20. Immunophenotyping panel for the identification and isolation of TIL. (a) Events were initially gated based on size and complexity, revealing a high presence of debris and cells of varying sizes due to tumor digestion. (b) As expected, cell viability within the lymphocyte gate was lower, reflecting the intensive processing required for isolation.(c) TIL-T and TIL-B populations were easily distinguished based on the expression of CD3 and CD19, respectively. (d) Further phenotyping included the evaluation of CD25, a marker associated with regulatory T cells (Treg), and CD45RO (e), which is linked to effector and memory T cell subsets. Notably, CD45RO+ T cells were predominant among TIL-T in the patients studied. (f) CD4+ and CD8+ TIL-T cells were further separated for detailed analysis, including lipid profiling. (g) Quantification of CD25+ and CD25- cell percentages within the CD3+ TIL population, shown as mean values from five independent tumor biopsies.

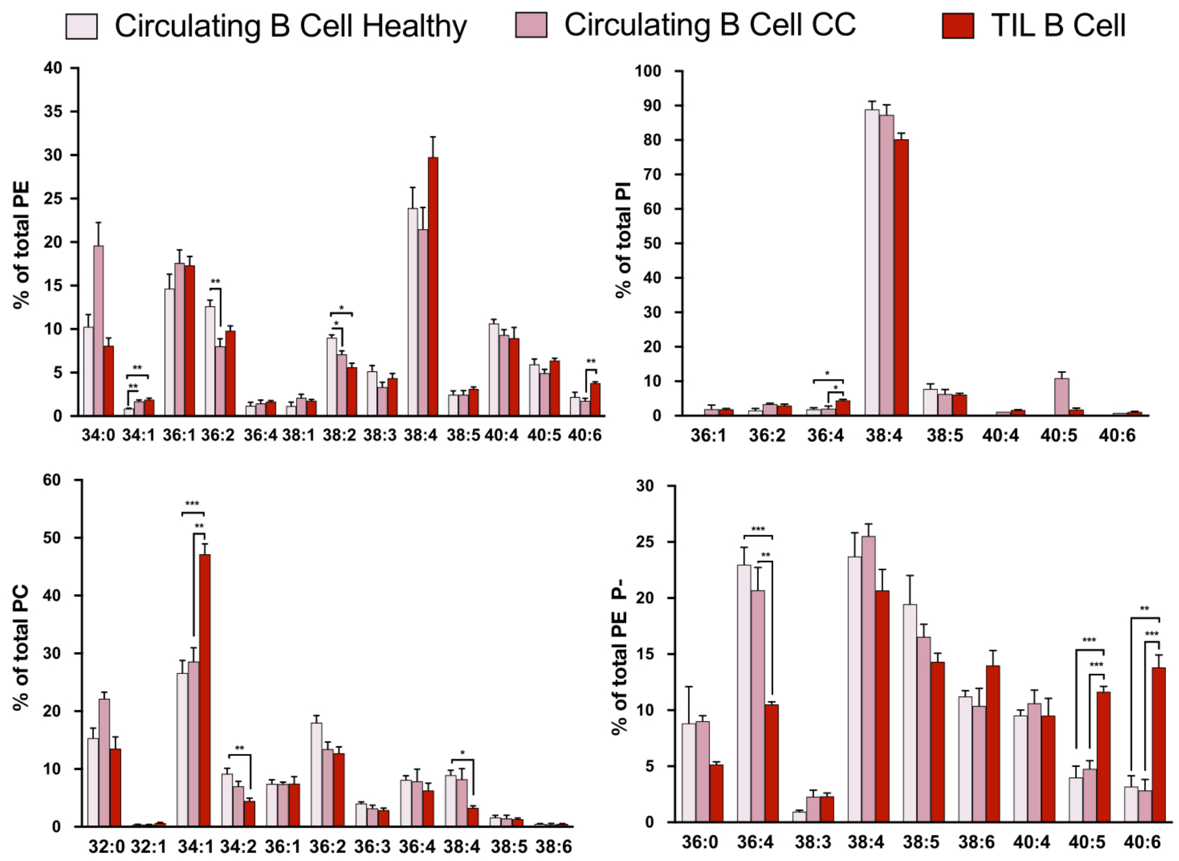

Figure S21. Lipid signature of circulating B Cells from healthy donors and CC patients and tumor- infiltrating B Cells (TIL-B). Bar diagrams showing the content within each lipid class in circulating B Cells cells obtained from healthy donors and CC patients and TIL-B. Values are represented as % for each lipid species within each of the main lipid classes detected on negative ion mode. Represent mean ± SEM, n = 5-7. Statistical significance was assessed by one-way ANOVA followed by Bonferroni post-test. * P < .05, ** P < .01, *** P < .001. Only species accounting for > 1% of the total membrane lipid class are included in the graph.

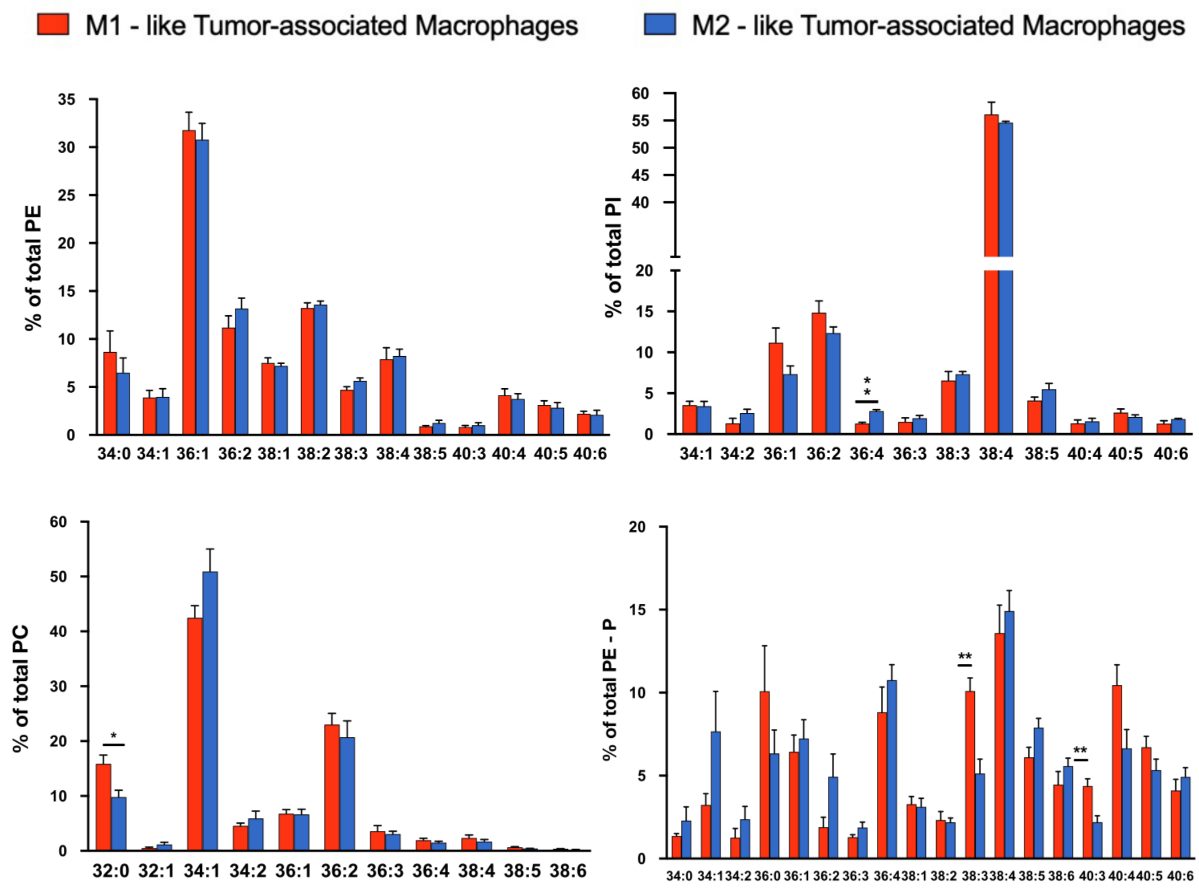

Figure S22. Lipid signature of M1 and M2-like tumor-associated macrophages (TAM). Bar diagrams showing the content within each lipid class in M1 and M2-like TAM. Values are represented as % for each lipid species within each of the main lipid classes detected on negative ion mode. Represent mean ± SEM, n = 5. Statistical significance was assessed using an unpaired Student's *t*-test, comparing the membrane lipid profiles M1 and M2-like TAM. The asterisk (*) indicates a significant difference between M1 and M2-like TAM. * P < .05, ** P < .01, *** P < .001. Only species accounting for > 1% of the total membrane lipid class are included in the graph.

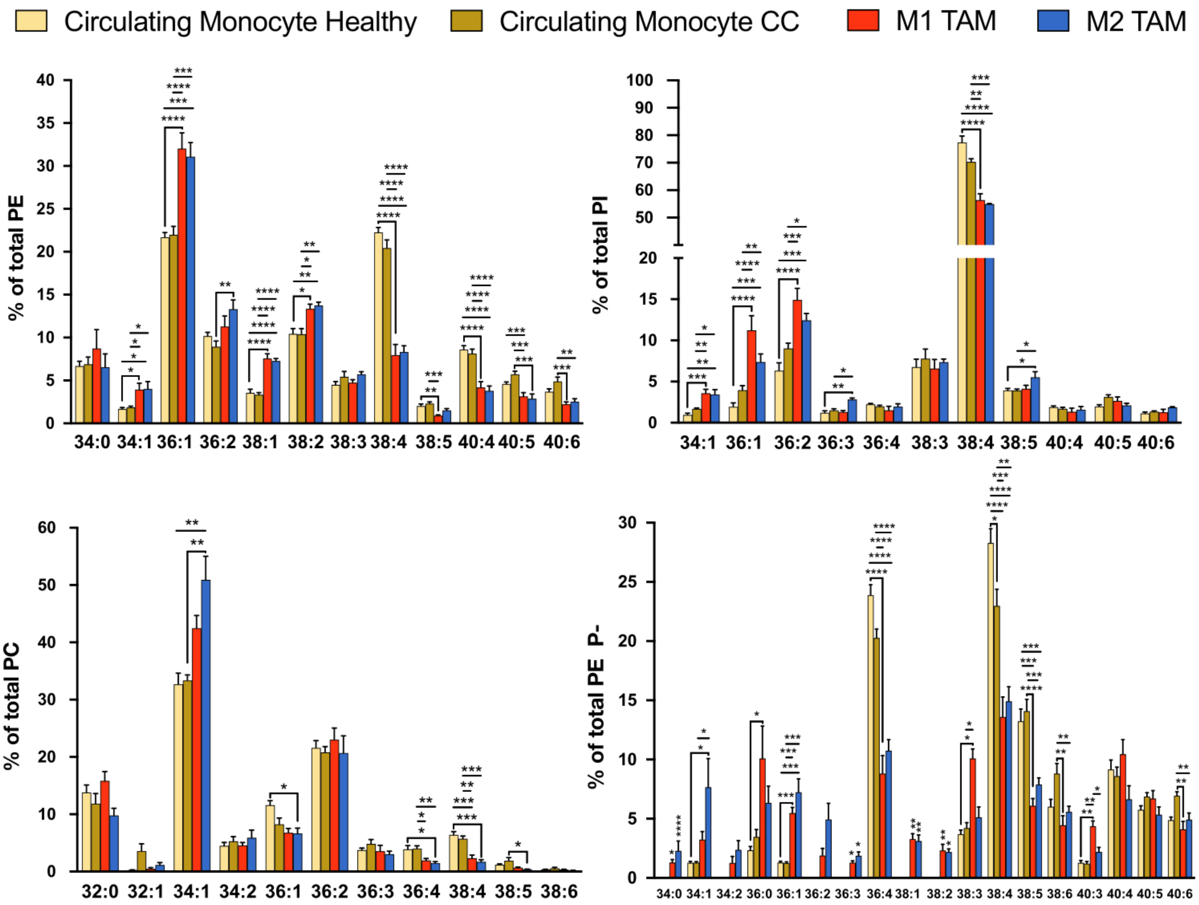

Figure S23. Lipid signature of circulating monocytes from healthy donors, CC patients, and M1 and M2-like tumor-associated macrophages (TAM). Bar diagrams showing the content within each lipid class in circulating monocytes from CC patients and M1 and M2-like tumor-associated macrophages (TAM). Values are represented as % for each lipid species within each of the main lipid classes detected on negative ion mode. Represent mean + SEM, n = 5. Statistical significance was assessed by one-way ANOVA followed by Bonferroni post-test. * $P < .05$, ** $P < .01$, *** $P < .001$. Only species accounting for > 1% of the total membrane lipid class are included in the graph.

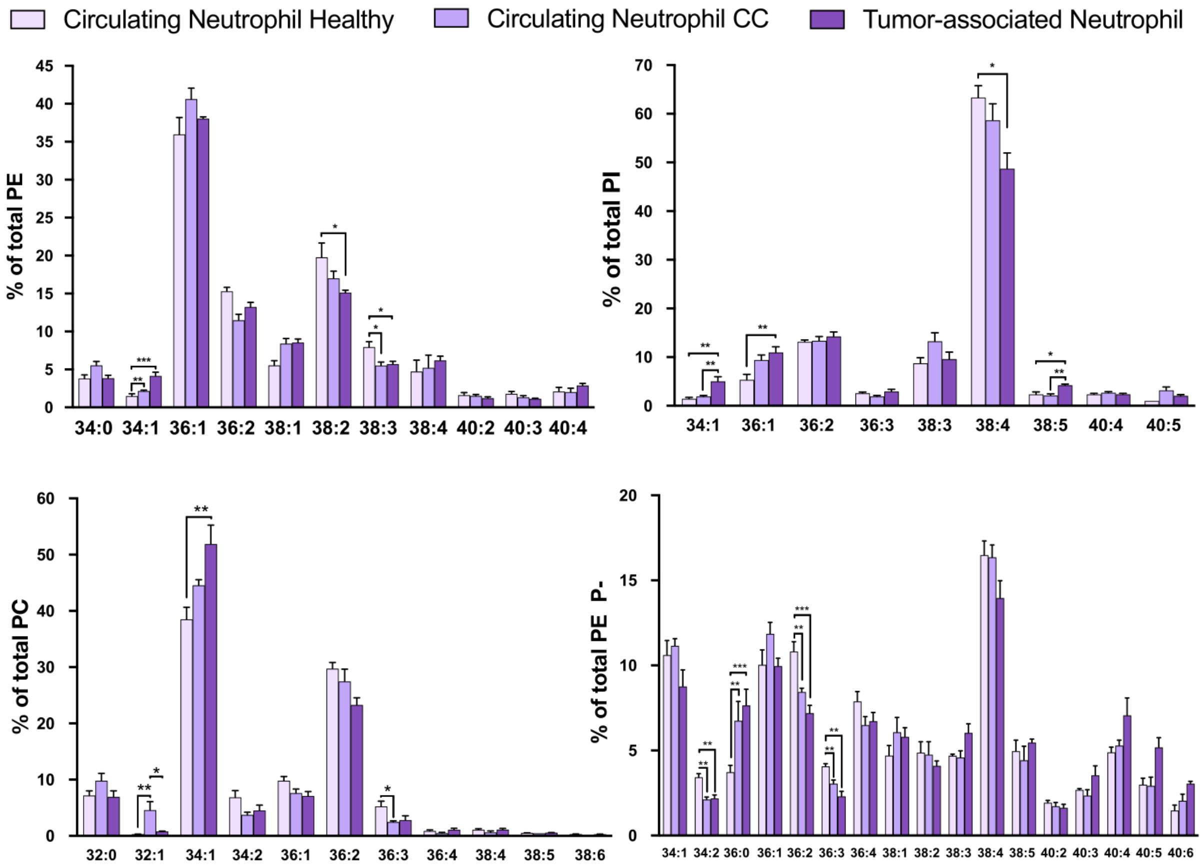

Figure S24. Lipid signature of circulating neutrophils from healthy donors and CC patients and tumor-associated neutrophils (TAN). Bar diagrams showing the content within each lipid class in circulating neutrophils from healthy donors and CC patients and tumor- associated neutrophils (TAN). Values are represented as % for each lipid species within each of the main lipid classes detected on negative ion mode. Represent mean + SEM, n = 5. Statistical significance was assessed by one-way ANOVA followed by Bonferroni post-test. * P < .05, ** P < .01, *** P < .001. Only species accounting for > 1% of the total membrane lipid class are included in the graph.

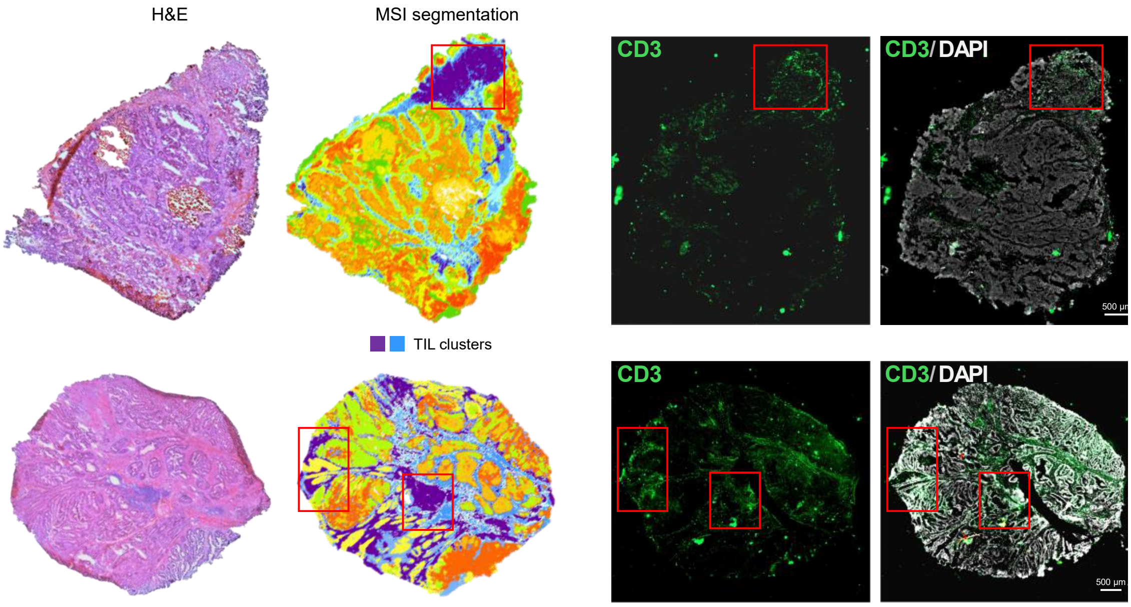

Figure S25. Immunofluorescence analysis of T cell infiltration in relation to histological and MSI lipidomic features. The figure shows representative images from two different tissue sections. The left panels present hematoxylin and eosin (H&E) staining and corresponding segmentation maps derived from mass spectrometry imaging (MSI)-based lipidomic analysis, which reveal molecularly distinct regions. Based on histological evaluation of the H&E sections, clusters corresponding to immune cell infiltration were identified and are represented by the purple and bright blue regions in the segmentation maps. The right panels show immunofluorescence staining performed on consecutive tissue sections, displaying $CD3^{+}$ T cells (green) and nuclei counterstained with DAPI (white). As the immunofluorescence was performed on adjacent sections, perfect histological alignment with the H&E and MSI panels is not expected. Images were acquired using a 10× objective and are shown as stitched composites. Scale bars represent 500 µm.

Table S3. Clinical and histopathological characteristics of patients.

**TOTAL, n**	20
**Age median (IQR)**	76 (67-81)
**Sex**	
Female, n(%)	10 (50%)
Male, n(%)	10 (50%)
**MMR protein expression loss, n (%)**	5 (25%)
**TNM, n (%)**	
pT2,	3 (15%)
pT3	13 (65%)
pT4	4 (20%)
**CRP median (IQR) (mg/dl)**	0.88 (0.15-3.09)
