# Supplementary material for: Advancing immune profiling in colon cancer through enhanced lipidomics of low‐input samples
Source: Clin Transl Med. 2025 Jul 11;15(7):e70399. doi: 10.1002/ctm2.70399 (PMC12246954; doi:10.1002/ctm2.70399)
Supplement: Supplementary file 2 — Supporting Information [file CTM2-15-e70399-s004.pdf]

Supplementary Materials and Methods

Advancing immune profiling in colon cancer through enhanced lipidomics of low-input samples

Running title: Cancer impacts on immune cells lipidome

Karim Pérez-Romero, Cristina Huergo-Baños, Albert Maimó-Barceló, Lucía Martín-Saíz, Catalina Crespí, Marco A. Martínez, Paloma de la Torre, Myriam Fernández-Isart, Daniel H. Lopez, José Andrés Fernández, Ramon M. Rodriguez, Gwendolyn Barceló-Coblijn.

Supplementary Materials and Methods

Patients and specimens

This study was approved by the Ethics Research Committee of the Balearic Islands (IB4568/21 PI), and all participants provided written informed consent. A total of 22 patients with colon cancer (12 women, 10 men) and 11 healthy donors (6 women, 5 men) were enrolled. Mean age of patients and donors was 72.9 and 30.6 years, respectively. Tissue samples were collected from individuals diagnosed with colon cancer adenocarcinoma undergoing surgical resection at the Department of Gastroenterology and General Surgery (University Hospital Son Espases). Inclusion criteria required a confirmed diagnosis of colon cancer adenocarcinoma, while exclusion criteria ruled out patients with concomitant colon diseases, prior neoadjuvant chemotherapy, other cancer diagnoses, or treatments related to other cancers. Specimens were obtained immediately post-surgery from the sigmoid colon (11), cecum (5), right colon (5), and transverse colon (1). None of the patients received chemotherapy or radiotherapy prior to resection. Fresh tissue was washed with phosphate-buffered saline, then either cryopreserved at -80°C for future use, or processed immediately for cell isolation. Clinical and histopathological characteristics of the patient with CC are summarized in Table S3; complete data were available for 20 of the 22 patients.

Flow sorting of circulating immune cells and sample preparation

Peripheral blood was drawn into lithium heparin tubes and mixed with PBS at a ratio of 1:1 v/v before separation by density gradient centrifugation (Ficoll™ solution, Sigma Aldrich). Subsequently, immune cells were isolated by Fluorescence-Activated Cell Sorting (FACS), using FACSAria Fusion (BD Biosciences). Circulating $CD4^+$ T cells were identified as $CD3^+$ (Biolegend, 1:100)/$CD4^+$ (BD Pharmigen, 1:100); $CD8^+$ T cells as $CD3^+$/$CD8^+$ (Biolegend, 1:100); NK cells as $CD3^-$/$CD56^+$ (Biolegend, 1:100); NKT

cells as $CD3^+/CD56^+$; monocytes as $CD14^+$ (BD Pharmigen, 1:40); neutrophils as $CD66b^+$ (Biolegend, 1:100), and B cells as $CD3^-/CD19^+$ (Biolegend, 1:100). Sorted immune cells (approx. 10^4 cells) were maintained in FACS buffer (PBS, 0.2% BSA, 2 mM EDTA) and centrifuged at 600 × g, 15 minutes at 4 °C. All sorted populations were collected at a minimum post-sort purity threshold of 95%, as ensured by stringent gating strategies. Dried cell pellets were saved at -80 °C.

Ex vivo stimulation assays

Immune cells from healthy donors were isolated as previously described. Naive T cells were isolated based on the surface expression of CCR7 (Biolegend, 1:100) and CD45RA (Biolegend, 1:100). Freshly sorted $CD4^+/CD8^+$ naive T cells were cultured in AIM V culture medium (Fisher Scientific) with IL-2 (Peprotech, 10 ng/ml) at 2.5×10^5 cells/mL, and stimulated with anti-CD3 (Biolegend, 12 μg/ml) and anti-CD28 (Biolegend, 2.2 μg/ml) monoclonal antibodies; 24 hours before naive T cell isolation, a 24-well plate was coated with the antibodies and incubated overnight at 4 °C before naive T cell deposition. Freshly sorted naive B cells, identified as $CD19^+/CD27^-$(Biolegend, 1:100)/IgD^+ (Biolegend, 1:100) were cultured in RPMI with L-glutamine medium (Labclinics) with 10% fetal bovine serum (FBS) premium (Labclinics), and penicillin-streptomycin (P/S) (Fisher Scientific, 1:100) at 5×10^5 cells/ml, and stimulated with sCD40 ligand (Peprotech, 1 μg/ml), IL-21 (Peprotech, 20 ng/ml), and AffiniPure IgA+IgG+IgM (Jackson ImmunoResearch Europe). Monocytes and neutrophils were cultured in RPMI with L-glutamine medium 10% FBS and P/S. Cells were treated with N-formyl-Met-Leu-Phe (Sigma-Aldrich, 43.75 ng/ml), phorbol 12-myristate 13-acetate (PMA) (Sigma-Aldrich, 12 ng/ml), interferon gamma (IFN-y) (Peprotech, 25 ng/ml), tumor necrosis factor alpha (TNF-α) (Peprotech, 20 ng/ml), and lipopolysaccharides from *E.coli* (LPS) (Sigma-Aldrich, 100 ng/ml). Finally, monocyte-derived macrophages were cultured in RPMI with L-glutamine medium 10% FBS and 1% P/S and polarized after GM-CSF (Peprotech, 100 ng/ml) or M-CSF (Peprotech, 180 ng/ml) treatment to M1 and M2-like macrophages, respectively. Stimulated cells and controls were harvested after 24 hours (neutrophils, monocytes), 72 hours (naive T and B cells), or 7 days (GM-CSF/M-CSF monocyte-derived macrophages) and saved at -80 °C.

Isolation of tumor-infiltrating immune cells

Fresh tumor specimens were rinsed in cold PBS, cut into small pieces (5-10 mm) and incubated in 10 volumes of digestion buffer (Hyclone Leibovitz L-15 media (Thermo Fisher Scientific)), 1% P/S, collagenase I, II, and IV (Thermo Fisher Scientific, 170, 57, and 170 μg/ml, respectively), hyaluronidase V (Merck Life Sciences, 30U/ml), and DNase I (Sigma-Aldrich, 10 mg/ml) for 60 minutes at 37 °C with continuous agitation. Tissue suspension was filtered through a 70 μm nylon mesh (SPL Life Sciences) to

harvest single cells. Cells were stained and sorted by FACS with the following markers: $CD3^+/CD4^+$ and $CD3^+/CD8^+$ for tumor-infiltrating T cells (TIL-T), $CD66b^+$ for tumor-associated neutrophils (TAN), and $CD3^-/CD19^+$ for tumor-infiltrating B cells (TIL-B). For tumor-associated macrophages (TAM) phenotyping, we established a Lin^-: CD66b, CD3, CD141 (Biolegend, 1:100), CD56, and CD19. Then, $Lin^-/CD45^+$ (Biolegend, 1:60)/$CD163^+$ (Biolegend, 1:100) were identified as M2-like TAM, and $Lin^-/CD45^+/CD163^-/CD11b^{high}$ (Biolegend, 1:100)/$CD11c^+$ (Biolegend, 1:100) as M1-like TAM. Sorted cells were washed with PBS and saved at -80 °C.

Sample Preparation and MALDI-MS imaging analysis

Dried cell pellets were thawed under controlled conditions (1 minute at 37 °C), resuspended in 10 µL of distilled water, and kept on ice to preserve molecular integrity. To achieve optimal confluence and enhance compatibility with MALDI-MS, the suspension was applied to 0.2% poly-L-lysine-coated glass slides using a sequential droplet layering technique, ensuring precise spatial confinement of 1 mm diameter droplets, promoting uniform sample density. For MALDI-MS imaging, tumor sections of 10 µm thickness were cryosectioned without cryoprotective agents or embedding material, maintaining native molecular profiles, and deposited onto indium tin oxide (ITO) glass slides (Hudson Surface Technology). All samples were cryopreserved at -80 °C to safeguard lipidomic integrity until imaging analysis. MBT (2-mercaptobenzothiazole, Sigma–Aldrich) and DAN (1,5-diaminonaphthalene, Sigma–Aldrich) were used as matrices for positive and negative ion detection, respectively, and deposited with the aid of an in-house sublimator.[1] Cell isolates were scanned in both positive and negative ion mode using a MALDI-LTQ-Orbitrap XL mass spectrometer (Thermo Fisher Scientific). Scanning range was 400–1,200 Da and mass resolutions of 30,000 at m/z = 400 Da. Spectra were analyzed with were normalized using a total ion current (TIC) algorithm and aligned using the Xiong method during the parsing stage using in-house built software based on Matlab (Mathworks).[2] Tumor sections were scanned in negative-ion mode, mass range of 300-1300 m/z, and 10 µm of lateral resolution, employing Tims ToF-Flex (Bruker) mass spectrometer (mass resolution 38000 at m/z 480). Spectra were analyzed using the dedicated software SCiLS Lab (Bruker). Briefly, MSI experiments were processed following standard data treatment, and peak intensity was normalized to total ion current. Regions of interest (ROIs) were extracted based image segmentation by bisecting k-means analysis with correlation distance and using approximately 450 features. The lipid maps database (www.lipidmaps.org), containing more than 33,000 lipid species, was used to assign lipid species based on m/z difference, with mass accuracy typically better than 5 ppm. Finally, lipid assigned data from tissue ROIs and isolated cells was normalized to the total lipid class % of molecular species, and further processed and plotted using Orange data mining software (v 3.35) and Prism 8 (Graphpad software, Inc).

Gene expression analysis

Transcriptomic data from previously published datasets GSE39596, GSE79828 and GSE123271 were analyzed to investigate the impact of immune cell activation on lipid gene expression. Raw data were normalized and log-transformed using the robust multi-array average method with the oligo R package (v 4.68). Differential expression was assessed using the limma R package (v 3.60.3). The R version used was 4.4.1. Genes with an absolute $\log_2$ fold change >1.5 and FDR <0.05 were deemed significant. Gene Set Enrichment Analysis (GSEA) was performed using GSEA software v4.3.2, focusing on pathways related to lipid metabolism from the Kyoto Encyclopedia of Genes and Genomes database: fatty acid biosynthesis (map00061), biosynthesis of unsaturated fatty acids (map01040), arachidonic acid metabolism (map00590), glycerophospholipid metabolism (map00564), ether lipid metabolism (map00565), and sphingolipid metabolism (map00600); using a total of 10,000 gene set permutations. Results are represented as a normalized enrichment score (NES).

Immunofluorescence staining

Tissue sections were fixed in 4% paraformaldehyde for 10 minutes at room temperature. After washing with PBS, sections were incubated in PBS containing 50 mM NH_4Cl for 5 minutes, washed again, and post-fixed in a 1:1 methanol-acetone solution. Permeabilization was performed with 0.1% Triton X-100 in PBS, followed by antigen retrieval in 1× EDTA-based unmasking solution (Cell Signaling Technology, #14747) for 25 minutes. After air-drying, sections were blocked in 5% BSA in PBS with 0.1% Tween-20 (PBS-T) for 1 hour at room temperature. Slides were incubated overnight at 4 °C with anti-CD3 epsilon antibody [SP7] (Abcam, 1:50) diluted in 0.1% BSA/PBS-T. After washing, sections were incubated with secondary antibodies (1:500) for 1 hour in the dark. Nuclei were stained with DAPI (1 µg/mL), followed by final washes and mounting with Fluoromount (Thermo Fisher Scientific). Slides were stored at 4 °C protected from light. Imaging was performed at the Advanced Microscopy Unit of IDISBA using a ZEISS LSM710 confocal microscope and Zen Blue software (ZEISS).

References

1. Fernández R, Garate J, Martín-Saiz L, et al. Matrix Sublimation Device for MALDI Mass Spectrometry Imaging. Anal Chem 2019;91:803–807.
2. Garate J, Lage S, Martín-Saiz L, et al. Influence of Lipid Fragmentation in the Data Analysis of Imaging Mass Spectrometry Experiments. J Am Soc Mass Spectrom 2020;31:517–526.
3. Yang C, Khanniche A, Dispirito JR, et al. Transcriptome Signatures Reveal Rapid Induction of Immune-Responsive Genes in Human Memory CD8+ T Cells. Sci Rep 2016;6:1–8. Available at: http://dx.doi.org/10.1038/srep27005.
4. Martínez-Llordella M, Esensten JH, Bailey-Bucktrout SL, et al. CD28-inducible transcription factor DEC1 is required for efficient autoreactive CD4+ T cell response. J Exp Med 2013;210:1603–1619.
